# A Comprehensive Peptidome Profiling Technology for the Identification of Early Detection Biomarkers for Lung Adenocarcinoma

**DOI:** 10.1371/journal.pone.0018567

**Published:** 2011-04-12

**Authors:** Koji Ueda, Naomi Saichi, Sachiko Takami, Daechun Kang, Atsuhiko Toyama, Yataro Daigo, Nobuhisa Ishikawa, Nobuoki Kohno, Kenji Tamura, Taro Shuin, Masato Nakayama, Taka-Aki Sato, Yusuke Nakamura, Hidewaki Nakagawa

**Affiliations:** 1 Laboratory for Biomarker Development, Center for Genomic Medicine, RIKEN, Yokohama, Japan; 2 CSK Institute for Sustainability, Ltd., Tokyo, Japan; 3 Laboratory of Molecular Medicine, Human Genome Center, Institute of Medical Science, The University of Tokyo, Tokyo, Japan; 4 Shimadzu Corporation, Kyoto, Japan; 5 Department of Molecular and Internal Medicine, Hiroshima University, Hiroshima, Japan; 6 Department of Urology, Kochi University School of Medicine, Nankoku, Japan; 7 Toppan Printing Co., Ltd., Tokyo, Japan; University of Houston, United States of America

## Abstract

The mass spectrometry-based peptidomics approaches have proven its usefulness in several areas such as the discovery of physiologically active peptides or biomarker candidates derived from various biological fluids including blood and cerebrospinal fluid. However, to identify biomarkers that are reproducible and clinically applicable, development of a novel technology, which enables rapid, sensitive, and quantitative analysis using hundreds of clinical specimens, has been eagerly awaited. Here we report an integrative peptidomic approach for identification of lung cancer-specific serum peptide biomarkers. It is based on the one-step effective enrichment of peptidome fractions (molecular weight of 1,000–5,000) with size exclusion chromatography in combination with the precise label-free quantification analysis of nano-LC/MS/MS data set using Expressionist proteome server platform. We applied this method to 92 serum samples well-managed with our SOP (standard operating procedure) (30 healthy controls and 62 lung adenocarcinoma patients), and quantitatively assessed the detected 3,537 peptide signals. Among them, 118 peptides showed significantly altered serum levels between the control and lung cancer groups (*p*<0.01 and fold change >5.0). Subsequently we identified peptide sequences by MS/MS analysis and further assessed the reproducibility of Expressionist-based quantification results and their diagnostic powers by MRM-based relative-quantification analysis for 96 independently prepared serum samples and found that APOA4 273–283, FIBA 5–16, and LBN 306–313 should be clinically useful biomarkers for both early detection and tumor staging of lung cancer. Our peptidome profiling technology can provide simple, high-throughput, and reliable quantification of a large number of clinical samples, which is applicable for diverse peptidome-targeting biomarker discoveries using any types of biological specimens.

## Introduction

Lung cancer is the leading cause of cancer death worldwide [1]. Smoking is still the leading risk factor for lung cancer, but recently the proportion of never smoker-related lung cancer is significantly increasing, although its cause or other risk factor(s) is unknown [2]. Lung cancer patients show the poor prognosis with an overall 5-year survival rate of only 15% [3]. One of the reasons for this dismal prognosis is no effective tools to detect it at an early stage and in fact only 16% of patients are diagnosed at their early stage of the disease [3]. Current screening methods such as chest X-ray or cytological examination of sputum have not yet shown their effectiveness in the improvement of mortality of lung cancer, whereas low dose helical CT have been proved to possess a potential to detect early-stage lung cancer and demonstrate 20% lower lung cancer mortality rate compared to chest X-ray screening [4]. On the other hand, serum biomarkers for lung cancer have been investigated to achieve early detection of the disease and improve clinical management of patients [5]. Nonetheless, their present clinical usefulness remains limited [6], [7]. CEA (carcinoembryonic antigen) and CYFRA (cytokeratin 19 fragment) are elevated in sera in a subset of lung cancer patients, and are clinically applied to monitor the disease status and evaluate the response to treatments. However, they are not recommended to use in clinical diagnosis and screening [8] because they are also elevated in certain non-cancerous conditions such as smoking and lung inflammation as well as in patients with other types of cancers. It is obvious that CEA and CYFRA do not have the sufficient power to apply for the screening of early-stage lung cancer. Hence, development of novel serum/plasma biomarkers applicable for lung cancer diagnosis is urgently required.

Recently monitoring the protein expression pattern in clinical specimens by proteomics technologies has offered great opportunities to discover potentially new biomarkers for cancer diagnosis. Various proteomic tools such as 2D-DIGE, SELDI-TOF-MS, protein arrays, ICAT, iTRAQ and MudPIT have been used for differential analysis of biological samples including cell lysates and blood to better understand the molecular basis of cancer pathogenesis and the characterization of disease-associated proteins [9]. In order to explore putative biomarkers in complicated biological samples, focused proteomics or targeted proteomics technologies have been utilized such as; phosphoprotein enrichment technologies IMAC [10], the cell-surface-capturing (CSC) technology [11], [12], glycan structure-specific quantification technology IGEL [13]. Most recently, to identify novel lung cancer biomarkers, Ostroff *et al.* reported the aptamer-based proteomic technology targeting 813 known proteins. Finally they selected 12 proteins which discriminated NSCLC from controls with 89% sensitivity and 83% specificity [14]. Thus targeted proteomics technologies such as the aptamer method would be applicable for the measurement of already known proteins, however could not be applied for the discovery of biomarkers targeting unknown proteins, post translational modifications, or biologically-processed polypeptides.

These methods can circumvent the technological limitations that currently prohibit the sensitive and high-throughput profiling of, in particular, blood proteome samples because of its high complexity and large dynamic range of proteins. The peptidome profiling technology addressed in the present study is one of the focused proteomics approaches targeting on biosynthetic fragments of proteins/peptides in blood, involving bioactive peptides and those non-specifically degraded by proteases or peptidases [15], [16].

So far more than 500 proteases/peptidases are known to be expressed in human cells [17], [18]. They function at almost all locations in the body including intracellular region, extracellular matrices, and in blood, involved in activation of other protein functions, degradation of cellular proteins, and notably tumor progression or suppression [19], [20], [21]. Indeed many matrix metalloproteases are overexpressed in various types of tumor cells, that facilitate construction of favorable micro-environment for tumor cells or promotion of metastasis[21]. Definitely these protease/peptidase activities should result in the production of digested peptide fragments well reflecting the tumor progression or tumor-associated responses. Thus peptidomic profiling of human serum or plasma is a promising tool for the discovery of novel tumor markers.

In this article, we extracted peptidome fractions (molecular weight <5,000 Da) from 92 individuals using the well-established and reproducible one-step peptidome enrichment method based on size exclusion chromatography (SEC) [22], [23] and provided them to the label-free mass spectrometric quantification analysis combined with the statistical analyses on Expressionist proteome server platform. Our rapid and simple peptidome enrichment procedure can circumvent both less reproducible peptidome extraction by such as ultrafiltration spin filters and prolonged sample preparation including immuno-depletion column chromatography, denaturing proteins, buffer exchange, ultrafiltration, and so on [16]. After quantitative comparison of 3,537 serum peptides among 92 cases in the lung cancer biomarker discovery, we further evaluated the accuracy of quantification results by another more reliable quantification method MRM (multiple reaction monitoring) technology using independently prepared 96 serum samples.

## Materials and Methods

### Serum samples

All human serum samples were obtained with informed consent from 122 patients with lung adenocarcinoma (stage I to IV) at Hiroshima University Hospital at the examination on admission. Serum samples as normal controls were also obtained with informed consent from 30 healthy volunteers who received medical checkup at Hiroshima NTT Hospital and 36 from Kochi University Hospital. Each consent above was given in writing. To circumvent undesirable degradation of proteins and peptides, all serum samples were collected and stored under unified SOP. Briefly, all venous blood specimens were collected with vacuum blood collection tubes TERUMO VP-P070K (TERUMO, Tokyo, Japan). After staying upright at ambient temperature for 60 minutes, serum fractions were separated with centrifugation at 1500 x *g* for 15 min (4°C) and immediately stored at −80 °C. One freeze-and-thaw procedure was permitted for any serum samples used in the present study. This study was approved by individual institutional ethical committees; The Ethical Committee of Yokohama Institute, RIKEN (Approval code: Yokohama H20-12), The Ethical Committee of Hiroshima University Hospital, and The Ethical Committee of Kochi University Hospital.

### Heat inactivation of sera and subsequent peptidome enrichment

All serum samples were freezed and thawed once and immediately incubated at 100 °C for 10 minutes after 4 times dilution with proteomics grade water. Following filtration with Spin-X 0.45 µm spin filters (Corning Incorporated, Corning, NY, USA), samples were loaded into 10/300 Superdex peptide column (GE Healthcare UK Ltd., Buckinghamshire, England) coupled with Prominence HPLC system (Shimadzu Corporation, Kyoto, Japan). The peptidome fraction was collected from 22 to 34 minutes in the constant flow of 100 mM ammonium acetate at 0.5 ml/min flow rate. The collected fractions were dried-up with Vacuum Spin Drier (TAITEC Co., Ltd., Saitama, Japan).

### LC/MS/MS analysis for the screening study

The dried peptide samples were resuspended in 2% acetonitrile with 0.1% trifluoroacetic acid and analyzed by QSTAR-Elite mass spectrometer (AB Sciex, Foster City, CA, USA) combined with UltiMate 3000 nano-flow HPLC system (DIONEX Corporation, Sunnyvale, CA, USA). Samples were separated on the 100 µm×200 mm tip-column (GL Sciences Inc., Tokyo, Japan), in which L-Column beads (Chemicals Evaluation and Research Institute, Tokyo, Japan) were manually loaded, using solvent A [0.1% formic acid, 2% acetonitrile] and solvent B [0.1% formic acid, 70% acetonitrile] with the multistep linear gradient of solvent B 5 to 55% for 95 minutes and 55 to 95% for 10 minutes at a flow rate 200 nl/min. The elute was directly analyzed with the 1 second MS survey (m/z 400–1800) followed by three MS/MS measurements on the most intense parent ions (30 counts threshold, +2 - +4 charge state, and m/z range 50–2000), using the “smart exit” setting (SIDA = 3.0, max accumulation time  = 2.0 sec.). Previously targeted parent ions were excluded from repetitive MS/MS acquisition for 40 seconds (100 mDa mass tolerance). The other parameters on QSTAR-Elite were shown as follows: DP  = 60, FP  = 265, DP2  = 15, CAD  = 5, IRD  = 6, IRW  = 5, Curtain gas  = 20, and Ion spray voltage  = 1600 V. The mass of each run was calibrated using three typical polysiloxane-derived background peaks: m/z  = 445.12003, 519.13882, and 667.17640. The resolution of mass spectra was around 20,000 at m/z  = 400. The primary data files (formatted as wiff and wiff.scan) from 92 clinical samples are available in a public repository site Proteome Commons (https://proteomecommons.org/). The MASCOT database search was performed on the Analyst QS 2.0 software (AB Sciex, Foster City, CA, USA). The MS/MS data was searched against the human protein database from SwissProt 57.4 (20,400 sequences) using the search parameters: Taxonomy  =  Homo sapiense, Enzyme  =  None, Fixed modifications  =  None, Variable modifications  =  Oxidation (Met), MS tolerance  = 50 ppm, and MS/MS tolerance  = 0.1 Da, with Mascot Automatic Decoy Search. Although Matrix Science recommends to use the Homology threshold for less-stringent criteria or Identity threshold providing almost same protein identification numbers with the criteria Expectation value <0.05 (http://www.matrixscience.com/help/interpretation_help.html), we accepted peptide identifications that satisfied both the false discovery rate (FDR) of peptide matches above identity threshold less than 5% and the Expectation value <0.05 in order to obtain more reliable identification of individual peptides than that from Mascot default criteria.

### Alignment of MS chromatogram planes and peak detection on Expressionist RefinerMS

The raw data files from QSTAR-Elite (.wiff and wiff.scan formatted) were directly loaded onto the Genedata Expressionist modules (Genedata AG, Basel, Switzerland). Genedata Expressionist worked on the in-house server system HP-DL380-G5 (Hewlett-Packard Development Company, Palo Alto, CA, USA) equipped with 16 GB memory, (72 GB×2) + (146 GB×25) RAID 0+1 hard disks, and SUSE Linux Enterprise Server 10 SP2 operating system, installed with Oracle 10 g ver. 10.2.0.4. software (Oracle Corporation, Redwood Shores, CA, USA). All MS chromatograms were smoothed with RT Window  = 3 scans in the Chromatogram Chemical Noise Subtraction Activity. To remove the background noise, a peak intensity is defined as follows.
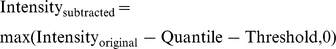



Here, values Quantile  = 50%, Intensity Threshold  = 15 cps were used. Furthermore signals satisfying at least one of the following criteria were considered as noise peaks and subtracted: RT Window >50 scans, Minimum RT Length  = 4 scans, or Minimum m/z Length  = 8 data points. Then MS chromatogram planes derived from 92 serum samples were accurately aligned using parameters: m/z Window  = 0.1 Da, RT Window  = 0.2 min, Gap Penalty  = 1, and RT Search Interval  = 5 min in the Chromatogram RT Alignment Activity. Next, the Summed Peak Detection Activity detected the peaks on a temporary averaged chromatogram with parameters as follows: Summation Window  = 5 scans, Overlap  = 50, Minimum Peak Size  = 4 scans, Maximum Merge Distance  = 10 data points, Gap/Peak Ratio  = 1, Method  =  curvature-based peak detection, Peak Refinement Threshold  = 5, Consistency Filter Threshold  = 0.8, Signal/Noise Threshold  = 1. Finally the two steps Summed Isotope Clustering Activity identified isotope patterns among 2D peaks, in which peaks identified as belonging to the same isotope pattern of a molecule were grouped into peak clusters. The first clustering was performed with the following criteria: Minimum Charge  = 1, Maximum Charge  = 10, Maximum Missing Peaks  = 0, First Allowed Gap Position  = 3, Ionization  = protonation, RT Tolerance  = 0.1 min, m/z Tolerance  = 0.05 Da, Isotope Shape Tolerance  = 10.0, and Minimum Cluster Size Ratio  = 1.2. The second clustering was performed with the same setting above, except for Minimum Cluster Size Ratio  = 0.6 and Reuse Existing Clusters  =  true. The information of all detected cluster peaks, including m/z, retention time, and intensity, was exported as ABS files.

### Label-free quantification and statistical analysis on Expressionist Analyst

The ABS files were loaded on the Expressionist Analyst module (Genedata AG, Basel, Switzerland). The peak intensity variation among 32 samples was normalized by fixing the median intensity of each sample at 10,000. Using the normalized intensity data, Student's t-test was performed between the normal group (n = 30) and lung cancer patients group (n = 62). The candidate biomarker peaks were extracted which showed *p*<0.01 and fold-change >5.0 between two groups. The candidates were further selected by Absent/Present Search to identify peaks with all-or-nothing detection pattern, which were detectable in 15 or all of 16 samples in one group and 1 or none of 16 samples in another group.

### Multiple Reaction Monitoring

Serum samples were processed with Superdex peptide column chromatography as described above before mass spectrometric analyses. The dried peptide samples were resuspended with 1 fmol/µl BSA tryptic digest solution in 2% acetonitrile, 0.1% trifluoroacetic acid and analyzed by 4000 QTRAP mass spectrometer (AB Sciex, Foster City, CA, USA) combined with Paradigm MS4 PAL nano-flow HPLC system (AMR Inc., Tokyo, Japan). Peptides were separated on the 100 µm×100 mm tip-column (GL Sciences Inc., Tokyo, Japan), in which L-Column ODS beads (Chemicals Evaluation and Research Institute, Saitama, Japan) were manually loaded. Using solvent A [0.1% formic acid, 2% acetonitrile] and solvent B [0.1% formic acid, 90% acetonitrile], the linear gradient of solvent B 2 to 100% for 10 minutes was configured at a flow rate 200 nl/min. 19 targeted peptide ions and 5 BSA-derived peptide ions were simultaneously monitored by the MRM mode in Analyst 1.5 software (AB Sciex, Foster City, CA, USA) in duplicate. The MRM transitions are shown in **Table S4**. The acquired MRM chromatograms were then smoothened and quantified with MultiQuant software (AB Sciex, Foster City, CA, USA). MRM peak areas in each sample were normalized as follows:
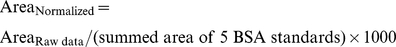



### Box plot analysis and ROC curve analysis

The averaged area of the duplicated MRM chromatogram peak corresponding to 19 candidate biomarker peptides was used to create box plot with R algorithm. For each study the box represents the middle half of the distribution of the data points stretching from the 25^th^ percentile to the 75^th^ percentile. The line across the box represents the median. The lengths of the lines above and below the box are defined by the maximum and minimum datapoint values, respectively, that lie within 1.5 times the spread of the box. Results of Student's t-test were included on the box plot. ROC curves were also depicted by R. The cut-off value was set at the point whose distance from the (sensitivity, specificity)  =  (1, 1) reached the minimum. The sensitivity (Sens), specificity (Spec), positive predictive value (PV+), negative predictive value (PV-), and are under the curve (AUC) were shown on each graph.

## Results

### The efficient enrichment of peptidome fractions from sera

**Figure 1:**
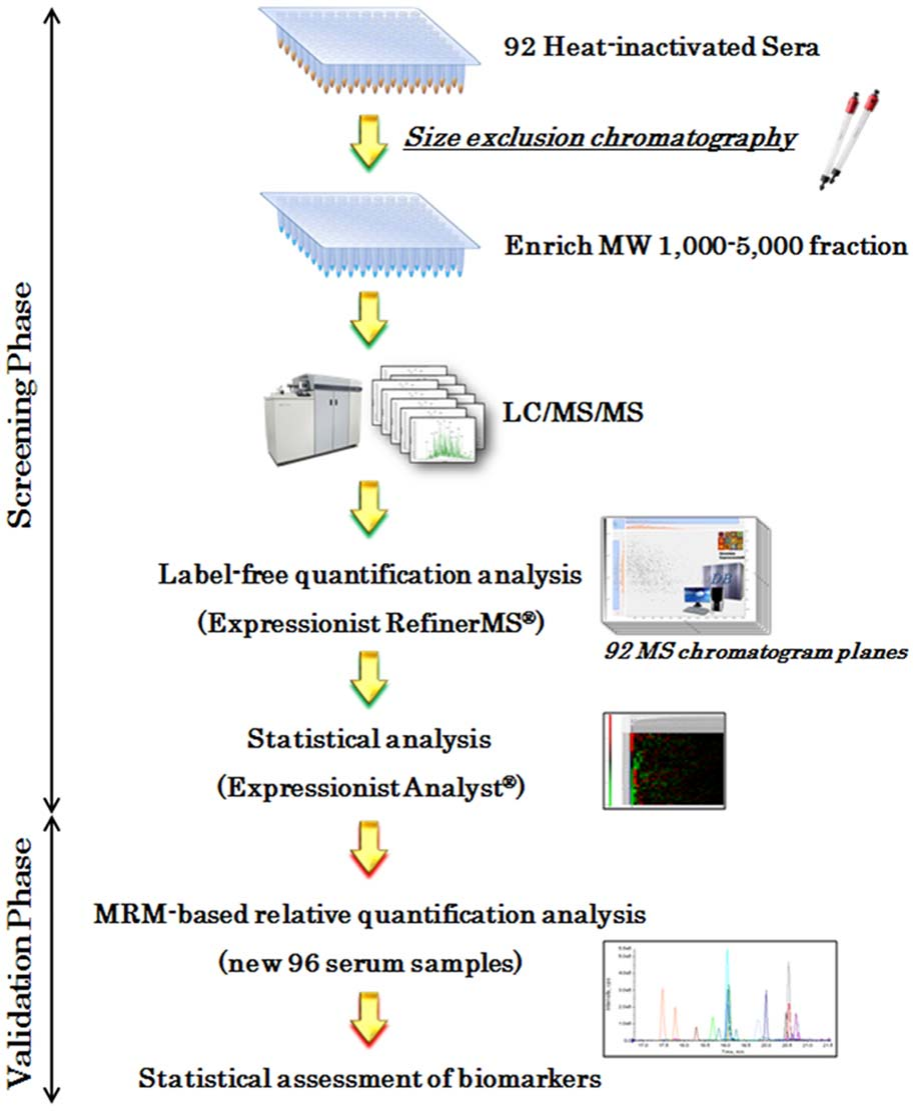
Schematic view of peptidome biomarker development workflow. In the screening phase, 92 serum samples were initially heat inactivated. The peptidome fractions enriched with gel filtration chromatography were analyzed with QSTAR-Elite LC/MS/MS. Following LC/MS data processing and label-free quantification analysis on the Expressionist RefinerMS module, candidate biomarkers were statistically extracted by the Expressionist Analyst module. In the validation phase, MRM experiments were performed to assess the applicability of 19 biomarker candidates using additional 96 serum samples.

**Figure 2:**
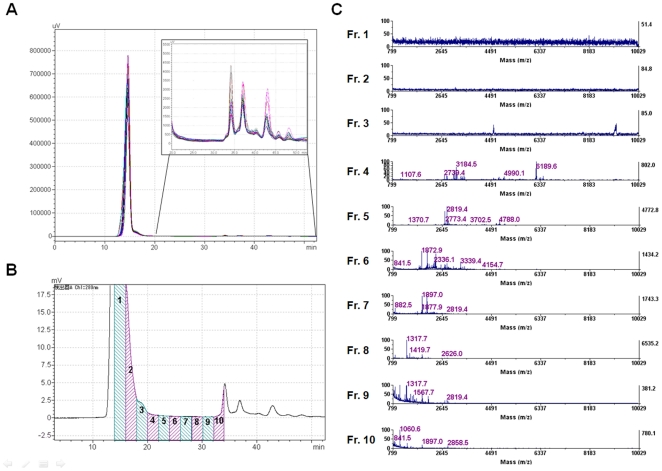
Simple and efficient enrichment of serum peptidome fractions by gel filtration chromatography. (*A*) The merged display of 16 independent spectra of gel filtration chromatography (280 nm UV absorbance). 10 µl each of serum sample was loaded. The upper right box shows the magnified view of the retention time range from 20 to 50 minutes. (*B*)(*C*) To evaluate the fractionation efficacy of Superdex Peptide 10/300 column, the elute was separated into 10 fractions and analyzed with MALDI-TOF mass spectrometer. The numbers of fractions in *B* correspond to the spectra numbers in *C*.

Since reproducible and accurate separation of the peptidome fraction from serum was essential for the effective screening of biomarkers, we optimized a simple gel filtration chromatography method (Fig. 1) and evaluated the peptide recovery. To avoid uncontrolled degradation of serum components arising from intact proteases and peptidases, all serum samples were immediately heated at 100 °C for 10 min after only one freeze-thaw procedure. Four-fold dilution of serum with water could eliminate the protein aggregation during heat inactivation even though the samples appeared slightly cloudy. Figure 2*A* shows the merged gel filtration HPLC chromatograms from 16 individual serum samples using the Superdex Peptide 10/300 column. The spectra illustrated highly reproducible separation of serum proteins and peptides. Then, the accuracy of size exclusion chromatography was assessed by analyzing 10 fractions (2 min each from retention time for the period of 14–34 min, Fig. 2*B*) with the MALDI-TOF-TOF mass spectrometer (Fig. 2*C*). As shown by the continuous MS spectra in Figure 2*C*, our gel filtration chromatography procedure allowed precise separation of serum proteins and peptides based on their molecular weights. Consequently we defined the fraction numbers 5 to 10 (corresponding to molecular weight 1,000 to 5,000) that should be focused in the further biomarker screening and validation studies.

### Label-free quantification-based peptide biomarker screening for lung cancer

**Figure 3:**
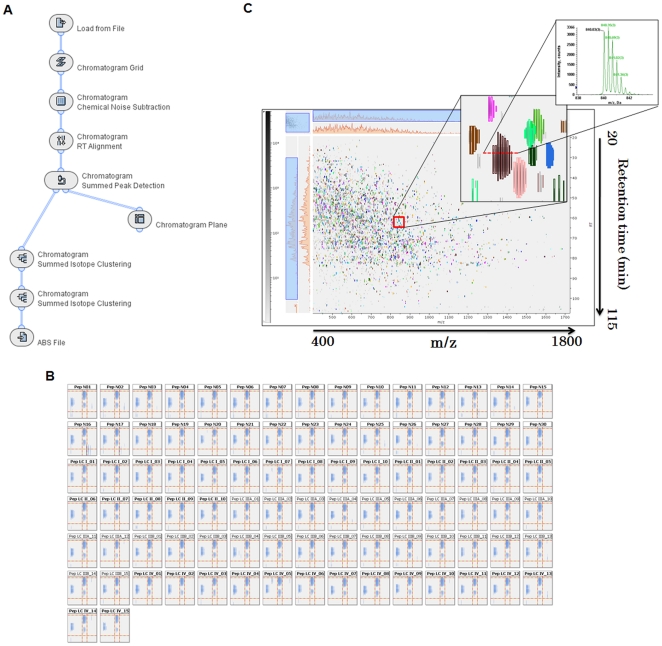
Rapid and accurate data processing for label-free quantification on the Expressionist RefinerMS module. (*A*) The total workflow used in the Expressionist RefinerMS module. Only 3 hours were needed to complete entire steps in this workflow on 92 LC/MS/MS data (each with 120 minutes LC gradient). (*B*) The representative area of m/z - retention time planes after RT alignment of 92 LC/MS/MS data. In each panel, three isotopic clusters and grid lines were displayed, showing highly exact alignments. (*C*) The MS chromatogram plane in which all data processing were completed. Finally, isotopic clusters derived from a single peptide were grouped into a colored cluster as shown in the middle panel. The far right panel shows the MS spectrum corresponding to the horizontal section view of a representative cluster.

To explore serum peptides which could be applied for early detection of lung cancer, we acquired quantitative peptidome profiles from 92 individuals (**Table S1**) including 62 lung cancer patients that consisted of 32 patients with an operable lung cancer (stage-I: n = 10, stage-II: n = 10, stage-IIIa: n = 12) and 30 lung cancer patients at an advanced stage (stage-IIIb: n = 15, stage-IV: n = 15) to identify candidate serum biomarkers for lung cancer. The serum samples were purified using gel filtration chromatography as described above and individually subjected to LC/MS/MS analyses using QSTAR-Elite mass spectrometer (Fig. 1). Subsequently 92 MS raw data were loaded and processed on the Expressionist RefinerMS module (Fig. 3*A*). Genedata Expressionist is an enterprise system for omics data management comprised of integrated software modules, which support the complete R&D processes involving data processing, statistical analysis, data management and result reporting. The technology-dependent modules for microarray data (Refiner Array), mass spectrometry (Refiner MS, used in the present study) and genomic profiling (Refiner Genome) allow highly-sophisticated data processing, quantification, visualization, and result exporting in any generally-used formats. Once all data are quantified and summarized, they can be seamlessly analyzed with the Genedata Analyst module employing various statistical analyses. This system initially made the MS chromatogram planes as shown in Figure 3*C*, and subtracted the instrument specific noises and chemical noises effectively. At the fourth step of the workflow in Figure 3*A*, the retention time (RT) grids on each MS chromatogram plane were perfectly aligned among these 92 samples (Fig. 3*B*), which allowed the solid quantification analysis of multiple samples. Subsequently, peaks were detected from temporarily averaged m/z-RT planes by the Chromatogram Summed Peak Detection Activity in order to avoid missing peak-location information even if the peaks were not detectable in particular planes. The detected isotopic peaks belonging to the same peptide signals were grouped into individual clusters that are displayed as colored rectangles in Figure 3*C*. A total of 12,396 non-redundant isotopic peak clusters with charge state +1 to +10 were detected from 92 serum samples. We then utilized 3,537 clusters with charge stage +2 to +10 for further statistical considerations in the Expressionist Analyst module, since singly-charged ions might include substantial proportion of non-peptide components such as chemicals.

**Figure 4:**
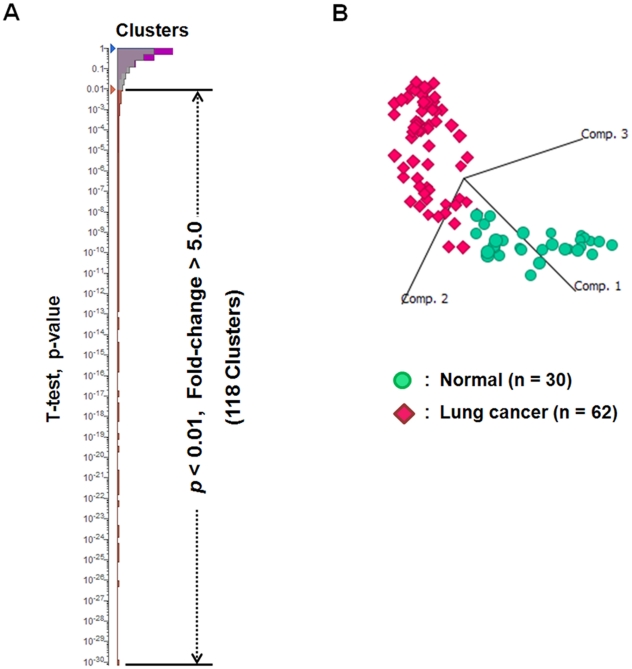
Statistical identification of candidate biomarkers for lung cancer. (*A*) The hierarchy chart of clusters (peptides) according to Student's t-test p-values (normal group vs. lung cancer group). 118 peptides satisfied the criteria of p<0.01 and fold change >5.0. (*B*) Principal component analysis using the values of 118 candidate biomarker peptides showed clear separation between control and lung cancer groups on the 3D plot. The proportion of variance described by the principal component 1, 2, or 3 was 66.9%, 15.0%, or 4.4%, respectively.

Student's t-test was applied to investigate the differences in their serum levels between the normal group (n = 30) and the lung cancer group (n = 62) (Fig. 4*A*). This analysis identified 118 candidate biomarker peptides (*p*<0.01 and fold-change of >5.0). Since the criteria of t-test were variable for the purpose of candidate selection, we used the threshold above just in order to define the highest priority group. The intensity distributions of these peptides were visualized with bar charts in Figure S1. The subsequent principal component analysis demonstrated that the values of 118 candidate biomarker peptides could explicitly separate control and lung cancer groups on the 3D plot using principal component 1, 2, and 3 (Fig. 4*B*). The proportion of variance described by the principal component 1, 2, or 3 was 66.9%, 15.0%, or 4.4%, respectively, indicating that illustrated components 1 to 3 could reflect 86.3% (the cumulative proportion) of quantitative information in this mass spectrometric screening analysis.

### Identification of peptide sequences by LC/MS/MS

Alongside the label-free quantification-based biomarker screening described above, the comprehensive peptide sequencing was performed by a combination of QSTAR-Elite LC/MS/MS analysis and MASCOT database search. Among 230,657 MS/MS queries from 92 serum samples, 5,382 peptides were successfully sequenced with MASCOT expectation value <0.05 (FDR of peptide matches above Identity threshold was 1.49%). After examining redundancy, 424 unique peptides were identified that corresponded to 106 proteins (**Table S2**). Regarding the 118 candidate peptides, 19 peptides were uniquely identified; 12 of them were found to be derivatives from fibrinogen alpha chain (FIBA), 4 from apolipoprotein A-IV (APOA4), and the remaining three peptides were turned out to be a fragment of amiloride-sensitive cation channel 4 (ACCN4), apolipoprotein E (APOE), and limbin (LBN) (**Table 1**).

**Table 1:** 19 lung cancer biomarker candidates.

Expressionist a					MASCOT b			
Cluster ID c	m/z	RT	z	t-test p-value d	Acc. e	start	end	Peptide sequence
Cluster_3187	551.8	64.1	2	1.54E-15	ACCN4	613	624	CPSLGRAEGGGV
Cluster_3858	750.9	60.3	2	7.85E-04	APOA4	271	283	ELGGHLDQQVEEF
Cluster_3444	629.8	52.2	2	9.41E-07	APOA4	268	284	GGHLDQQVEEF
Cluster_3661	689.8	75.3	2	8.52E-08	APOA4	260	284	GNTEGLQKSLAELGGHLDQQVEEFR
Cluster_3498	643.3	65.7	2	6.08E-05	APOA4	288	304	SLAELGGHLDQQVEEFR
Cluster_2454	756.4	65.6	3	2.93E-03	APOE	194	214	TVGSLAGQPLQERAQAWGERL
Cluster_248	768.8	53.0	2	6.41E-23	FIBA	1	16	ADSGEGDFLAEGGGVR
Cluster_126	432.7	62.6	2	3.07E-22	FIBA	7	15	DFLAEGGGV
Cluster_159	510.7	49.9	2	5.75E-25	FIBA	7	16	DFLAEGGGVR
Cluster_240	733.3	56.5	2	3.80E-15	FIBA	2	16	DSGEGDFLAEGGGVR
Cluster_166	525.7	62.8	2	2.99E-25	FIBA	5	15	EGDFLAEGGGV
Cluster_3342	603.8	50.7	2	8.17E-27	FIBA	5	16	EGDFLAEGGGVR
Cluster_2872	461.2	61.8	2	3.31E-12	FIBA	6	15	GDFLAEGGGV
Cluster_174	539.3	52.3	2	4.10E-15	FIBA	6	16	GDFLAEGGGVR
Cluster_180	554.2	63.5	2	5.37E-22	FIBA	4	15	GEGDFLAEGGGV
Cluster_207	632.3	52.1	2	1.98E-21	FIBA	4	16	GEGDFLAEGGGVR
Cluster_196	597.8	63.2	2	4.44E-24	FIBA	3	15	SGEGDFLAEGGGV
Cluster_221	675.8	52.3	2	2.22E-22	FIBA	3	16	SGEGDFLAEGGGVR
Cluster_135	453.2	39.0	2	2.81E-24	LBN	306	313	FLLSLVLT

^*a*^Information acquired from the Expressionist RefinerMS or the Analyst module.

^*b*^Information acquired from MASCOT database search.

^*c*^Each ID corresponds to that in the bar chart (Fig. S1).

^*d*^Shown is the p-value of t-test between normal group and lung cancer group.

^*e*^UniProt Accession Number.

### MRM-based validation experiment for 19 candidate biomarker peptides

**Figure 5:**
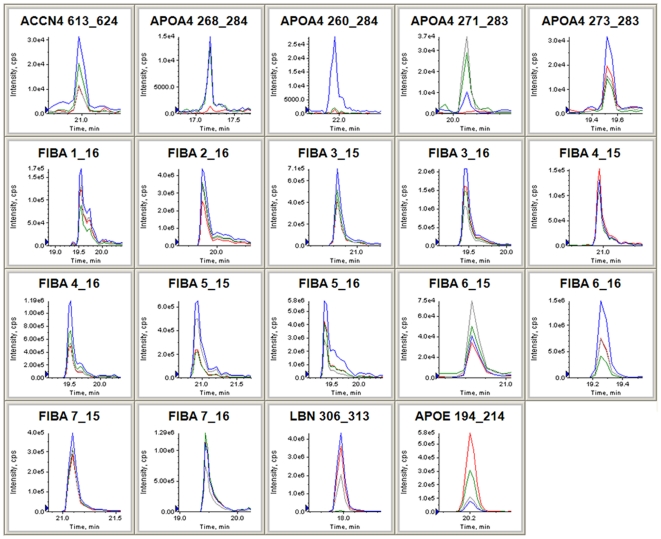
Selection and confirmation of the optimum MRM transitions for 19 candidates. Four pairs of precursor m/z and fragment m/z (Q1/Q3 channels) were set as MRM transitions for each peptide. The blue, red, green, or gray MRM chromatogram monitored the fragment ion which showed the 1^st^, 2^nd^, 3^rd^, or 4^th^ most intense peaks in QSTAR-Elite LC/MS/MS analysis, respectively.

**Figure 6:**
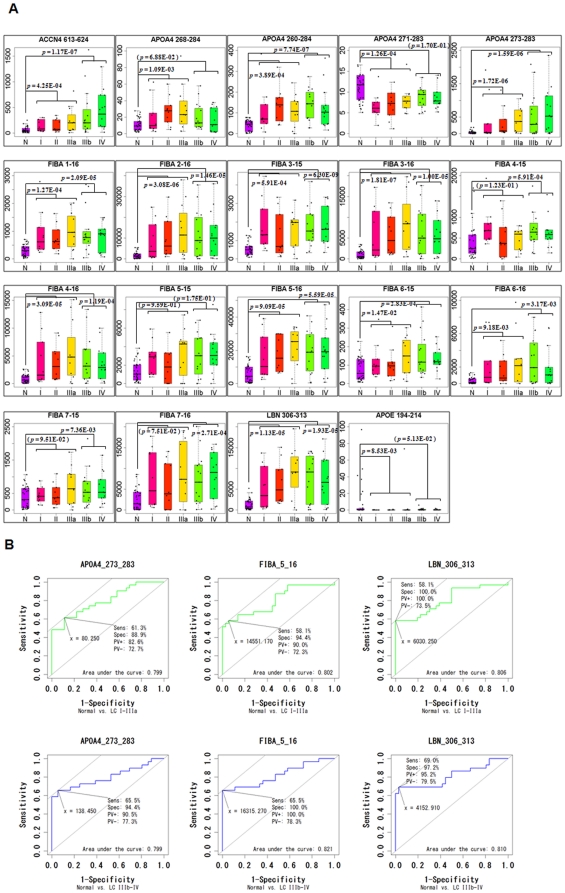
Statistical assessment of MRM-based validation experiments. (*A*) Box plots representing the stage-dependent distributions of serum levels of the 19 candidate biomarkers. The p-values from t-test between “normal group (n = 36) and lung cancer stage-I, II, and IIIa (n = 30)” or “normal group (n = 36) and lung cancer stage-IIIb and IV (n = 30)” are shown. The p-values that did not show significant differences were provided in parentheses. N: normal group, I, II, IIIa, IIIb, and IV: lung cancer stage-I, II, IIIa, IIIb, and IV group, respectively. (*B*) ROC curves for APOA4 206–284, FIBA 2–16, and LBN 306–313 were depicted by R. The green or blue graph shows comparison of “normal group (n = 36) and lung cancer stage-I, II, and IIIa (n = 30)” or “normal group (n = 36) and lung cancer stage-IIIb and IV (n = 30)”, respectively. The cut-off value was set at the point whose distance from the (sensitivity, specificity)  =  (1, 1) reached the minimum. The sensitivity (Sens), specificity (Spec), positive predictive value (PV+), negative predictive value (PV-), and area under the curve (AUC) were shown on each graph.

To assess the quantitative reproducibility of the label-free quantification results in our single-run screening analysis, as well as the clinical usefulness of the 19 candidate biomarkers, we conducted further validation studies by multiple reaction monitoring (MRM) technology using 96 additional serum samples (**Table S1**). For designing the optimum MRM transitions specific to the 19 candidate peptides, the m/z values of precursor ions detected in the screening phase were set as Q1 channels and those of four most intense fragment ions were selected from each MS/MS spectrum for Q3 channels (Fig. S2 and Table S3). Hence, a total of 76 MRM transitions were simultaneously monitored by 4000 QTRAP mass spectrometry using a serum peptidome sample (Fig. 5). We then determined the specific eluting retention time for each candidate peptide and selected the optimum MRM transitions showing the highest MRM chromatogram peak out of four transitions for each peptide (Table S4). In our observations, only two peptides (FIBA 3–16 and FIBA 5–16) showed the identical orders of fragment ion intensities between QSTAR-Elite and 4000 QTRAP systems as shown in Figure 5. We further performed MRM-based relative quantification analysis using 36 normal controls and 60 lung cancer samples in duplicated experiments. The serum levels of 19 candidate biomarker peptides were calculated on the basis of normalized and averaged MRM chromatogram peak areas and displayed with box plots (Fig. 6*A*). To evaluate the efficacy of these candidates for early detection of lung cancer, we compared the earlier-stage lung cancer group (stage-I, II, and IIIa) with the normal group by Student's t-test. The results revealed that 15 out of 19 candidate peptides showed significant differences in their serum levels between the two groups, while 4 peptides (FIBA 4–15, FIBA 5–15, FIBA 7–15, and FIBA 7–16) showed no significant differences. Concerning the comparison between the normal group and the advanced-stage lung cancer group (stage-IIIb and IV), similarly 4 peptides (APOA4 268–284, APOA4 271–283, FIBA 5–15, and APOE 194–214) did not satisfy the criterion of *p*<0.05. Hence, we considered that the remaining 12 peptides are likely to be more promising biomarkers for lung cancer diagnosis. We next assessed the sensitivity and specificity of the 19 biomarkers for lung cancer diagnosis by ROC curve analysis (Fig. 6*B* and Fig. S3). The cut-off value was set at the point whose distance from the (sensitivity, specificity)  =  (1, 1) reached the minimum. Given the value of sensitivity to detect lung cancer at an earlier stage, FIBA 6–15 (87.1%), APOA4 273–283 (61.3%), FIBA 5–16 (58.1%), and LBN 306–313 (58.1%) appeared to be the good biomarker candidates. However although the specificity of APOA4 273–283, FIBA 5–16, and LBN 306–313 were remarkably higher (88.9%, 94.4%, and 100%, respectively, Fig. 6*B*), FIBA 6–15 showed relatively lower specificity (44.4%) and the area under the curve (0.641). By integrating the results from t-test and ROC curve analysis, the 3 candidates shown in Figure 6*B* were considered as the most promising peptide biomarkers for early detection of lung cancer.

## Discussion

Even though recent mass spectrometry instruments have allowed measurements of peptide mixtures at high sensitivity [24], enrichment of targeted proteins/peptides is still indispensable to achieving detection and identification of serum components in limited amounts of biological materials. In this sense, the methodology to purify preanalytical samples without loss of targeted components is crucial. From this point of view, the previous peptidome profiling technologies, such as SELDI-TOF-MS coupled with ProteinChip arrays or MALDI-TOF-MS analysis of ClinProt magnetic beads-purified samples, covered only limited spectra of serum peptidome. Most of studies utilizing ion-exchange selection or reversed phase extraction of peptidome on ProteinChip arrays [25], [26], [27] or magnetic beads [28], [29] allowed at most 200 peak detections within the mass range 1,000 to 20,000. Meanwhile our peptidome profiling technology consisting of gel-filtration chromatography, custom-made high resolution C18 tip-column, QSTAR-Elite mass spectrometer, and Expressionist proteome server platform analysis enabled us to detect 12,396 non-redundant molecules with charge state of +1 to +10. The number of detected peaks here denoted the enormous advantage of our methodology for the analytical comprehensiveness compared to other existing methods. Although we focused on serum peptides involved in 3,537 clusters with charge stage of +2 to +6 in this study, 12,396 clusters might include non-peptide serum components such as metabolites, which should be also valuable for biomarker screening. Additionally, regarding the capacity of sample numbers to be analyzed simultaneously, the Expressionist server platform has a potential to handle a larger number of clinical samples. Because we in fact needed only less than an hour to process 92 LC/MS/MS data in the Refiner MS module (Fig. 1*A*), a comprehensive analysis of up to 1,000 cases would be feasible in a day. Hence our peptidome profiling technology provides the outstanding features of data comprehensiveness and quantitative performance, which absolutely fit the in-depth screening of novel biomarkers from clinical samples such as serum and plasma compared to previous technologies described above, whereas estimating actual dynamic range of detected peptide concentrations would be needed by,for instance, MRM-based absolute quantification analysis in the future. It could be tailored to many diagnostic and pharmaco-dynamic purposes as comprehensive interpretations of catalytic and metabolic activities in body fluids or tissues.

By using this technology, we finally identified 19 serum peptides as candidate lung cancer biomarkers (Table 1). The subsequent MRM-based validation experiments and t-test resulted in the confirmation of 12 candidates as reliable lung cancer biomarkers (Fig. 6*A*). Eight of them were fragments derived from fibrinopeptide A (FPA) which is N terminally cleaved product from fibrinogen α (FIBA). In fact, both our screening and validation results suggested that all of these eight FPA fragments were potential lung cancer-associated biomarkers showing the significant increase of concentrations in lung cancer patients' sera. However, since anomalous turnover of FPA was previously reported in several other diseases including gastric cancer [30], diabetic nephropathy [31], coronary heart disease [32], and others, these 8 FPA fragments could not be defined as lung cancer-specific biomarkers. The other two candidates were generated from apolipoprotein A-IV (APOA4). APOA4 protein itself was already identified as an up-regulated biomarker for ovarian cancer [33], whereas this was also known to be regulated by nutritional and metabolic stress [34]. But both quantitative information and physiological functions of endogenously-processed APOA4 peptides in human serum were still unknown. Interestingly, the APOA4 273–283 fragment demonstrated pathological stage-dependent up-regulation in lung cancer patients' sera, while the two-residue longer fragment APOA4 271–283 was significantly decreased in lung cancer samples (Fig. 6*A*). This indicates the existence of lung cancer-associated endo- or exopeptidases responsible for the cleavage at the C-terminus of APOA4 a.a. 272. Additional two candidate biomarkers, LBN 306–313 and ACCN4 613–624, derived from limbin (LBN) and amiloride-sensitive cation channel 4 (ACCN4) proteins, were reported as cellular membrane proteins. LBN is also known as Ellis-van Creveld syndrome 2 (EVC2) that is expressed in the heart, placenta, lung, liver, skeletal muscle, kidney and pancreas. Defects in LBN (EVC2) are a cause of acrofacial dysostosis Weyers type (WAD, also known as Curry-Hall syndrome) [35]. ACCN4 is a newly identified member of the acid-sensing ion channel family expressed in pituitary gland and weakly in brain [36]. Neither of them was detected in serum previously. Since our study provided the first evidence of LBN 306–313 and ACCN4 613–624 detection in human serum, further analysis of physiological functions and measurement in other diseases should be required for the proper use in clinical lung cancer diagnosis. Hence, the three candidate biomarkers illustrated in Figure 6*B* (APOA4 273–283, FIBA 5–16, and LBN 306–313) were individually considered as clinically useful biomarkers for both early detection and tumor staging of lung cancer, however, integrative measurement of biomarkers such as Figure 4*B* would provide more accurate diagnosis, that could be achievable by MRM-based diagnostic approaches in the future. Consequently the sensitivity of these biomarkers was higher than the currently-used screening biomarker CEA especially at even stage-I or II [8], indicating that new biomarkers addressed in this study had great potential to realize the early detection system for lung cancer. However further validation experiments using high risk groups of lung cancer as the controls (such as heavy smokers or COPD patients) will be necessary to prove the specificity and clinical usefulness of our biomarkers because more practical target population of the early diagnosis of lung cancer should be them rather than healthy individuals.

Finally we grasped the birds-eye view of human peptidome as a snapshot of the specific disease state. We are recently willing to use our peptidome profiling technology to establish an in-house quantitative serum/plasma peptidome database and contribute to the worldwide efforts such as Peptide Atlas (http://www.peptideatlas.org/). This framework would represent a new insight of protease/peptidase activities reflecting a clinical status at a specific time-point of disease and provide essential resources for next-generation extracorporeal diagnostic systems based on mass spectrometry. We therefore hope that researchers at global sites would utilize the peptidome profiling method addressed here and share data to construct mutually beneficial networks and databases which could contribute to the development of future diagnostic technologies worldwide.

## Supporting Information

Figure S1 The bar charts illustrating the quantitative screening results for 19 candidates. The normalized peak intensities of 118 candidate biomarker peptides were calculated from 92 serum samples and displayed with bar charts.
(TIF)

Figure S2 MS/MS spectra used for the construction of MRM transitions and peptide identification. All MS/MS spectra were acquired with QSTAR-Elite mass spectrometer in the screening phase (the upper panels). The 1^st^, 2^nd^, 3^rd^, or 4^th^ most intense peaks in each MS/MS spectrum were used for the optimization of MRM transitions (Fig. 5) The middle and the lower panels show the identified fragment ions in MASCOT database search. The ion scores and Expectation values were also indicated in the lower panels.
(TIF)

Figure S3 ROC curves for 19 lung cancer biomarker candidates were depicted by R. The green or blue graph shows comparison of “normal group (n = 36) and lung cancer stage-I, II, and IIIa (n = 30)” or “normal group (n = 36) and lung cancer stage-IIIb and IV (n = 30)”, respectively. The cut-off value was set at the point whose distance from the (sensitivity, specificity)  =  (1, 1) reached the minimum. The sensitivity (Sens), specificity (Spec), positive predictive value (PV+), negative predictive value (PV-), and area under the curve (AUC) were shown on each graph.
(TIF)

Table S1 (DOC)

Table S2 (DOC)

Table S3 (DOC)

Table S4 (DOC)
