# Supplementary figures and images for: A Comprehensive Peptidome Profiling Technology for the Identification of Early Detection Biomarkers for Lung Adenocarcinoma
Source: PLoS One. 2011 Apr 12;6(4):e18567. doi: 10.1371/journal.pone.0018567 (PMC3075260; doi:10.1371/journal.pone.0018567)

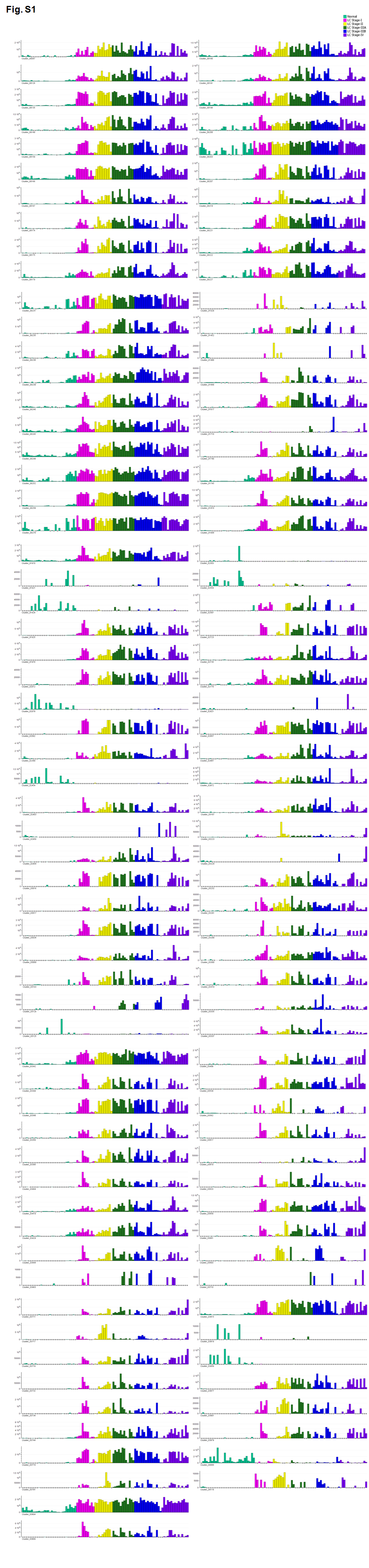

Supplement: Figure S1 — The bar charts illustrating the quantitative screening results for 19 candidates. The normalized peak intensities of 118 candidate biomarker peptides were calculated from 92 serum samples and displayed with bar charts. (TIF) [file pone.0018567.s001.tif]

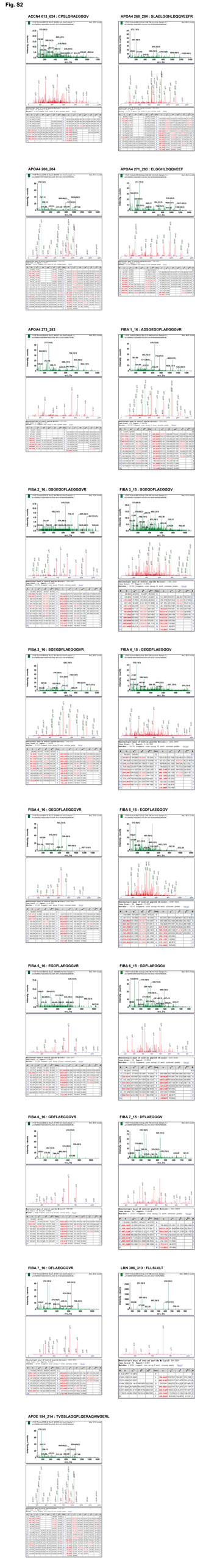

Supplement: Figure S2 — MS/MS spectra used for the construction of MRM transitions and peptide identification. All MS/MS spectra were acquired with QSTAR-Elite mass spectrometer in the screening phase (the upper panels). The 1st, 2nd, 3rd, or 4th most intense peaks in each MS/MS spectrum were used for the optimization of MRM transitions (Fig. 5) The middle and the lower panels show the identified fragment ions in MASCOT database search. The ion scores and Expectation values were also indicated in the lower panels. (TIF) [file pone.0018567.s002.tif]

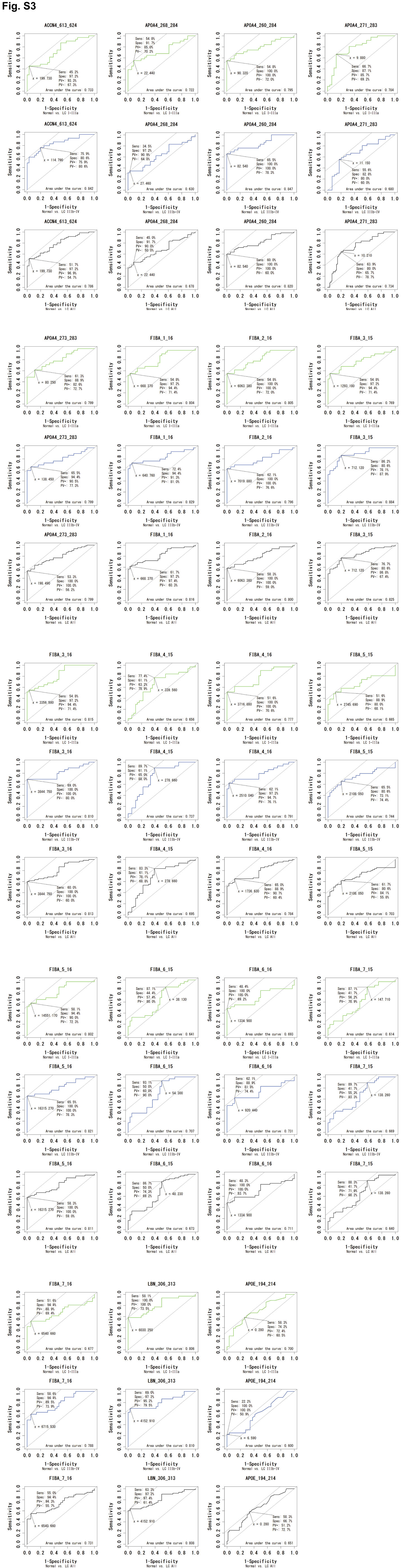

Supplement: Figure S3 — ROC curves for 19 lung cancer biomarker candidates were depicted by R. The green or blue graph shows comparison of “normal group (n = 36) and lung cancer stage-I, II, and IIIa (n = 30)” or “normal group (n = 36) and lung cancer stage-IIIb and IV (n = 30)”, respectively. The cut-off value was set at the point whose distance from the (sensitivity, specificity) = (1, 1) reached the minimum. The sensitivity (Sens), specificity (Spec), positive predictive value (PV+), negative predictive value (PV-), and area under the curve (AUC) were shown on each graph. (TIF) [file pone.0018567.s003.tif]
