# Supplementary material for: A Comprehensive Peptidome Profiling Technology for the Identification of Early Detection Biomarkers for Lung Adenocarcinoma
Source: PLoS One. 2011 Apr 12;6(4):e18567. doi: 10.1371/journal.pone.0018567 (PMC3075260; doi:10.1371/journal.pone.0018567)
Supplement: Table S1 — (DOC) [file pone.0018567.s004.doc]

**Table S1**

*92 serum samples used in the peptidome biomarker screening.*

| Group | Case number (Male/ Female) | Averaged age |
| --- | --- | --- |
| Healthy controls | 30 (20 / 10) | 56.7 |
| Lung adenocarcinoma stage-I | 10 (7 / 3) | 63.7 |
| Lung adenocarcinoma stage-II | 10 (6 / 4) | 63.1 |
| Lung adenocarcinoma stage-IIIa | 12 (8 / 4) | 63.6 |
| Lung adenocarcinoma stage-IIIb | 15 (10 / 5) | 64.0 |
| Lung adenocarcinoma stage-IV | 15 (10 / 5) | 63.7 |

*96 serum samples used in the validation step.*

| Group | Case number (Male/ Female) | Averaged age |
| --- | --- | --- |
| Healthy controls | 36 (26 / 10) | 63.6 |
| Lung adenocarcinoma stage-I | 10 (7 / 3) | 64.2 |
| Lung adenocarcinoma stage-II | 10 (7 / 3) | 62.1 |
| Lung adenocarcinoma stage-IIIa | 11 (7 / 4) | 66.0 |
| Lung adenocarcinoma stage-IIIb | 15 (10 / 5) | 64.3 |
| Lung adenocarcinoma stage-IV | 14 (10 / 4) | 66.4 |
