# Supplementary material for: A Comprehensive Peptidome Profiling Technology for the Identification of Early Detection Biomarkers for Lung Adenocarcinoma
Source: PLoS One. 2011 Apr 12;6(4):e18567. doi: 10.1371/journal.pone.0018567 (PMC3075260; doi:10.1371/journal.pone.0018567)
Supplement: Table S2 — (DOC) [file pone.0018567.s005.doc]

**Table S2**

*All of identified 424 peptides by LC/MS/MS from 92 serum samples.*

*Peptide hits with MASCOT Expectation value < 0.05 were shown.*

| **Acc.** | **Name** | **m/z** | **z** | **Start** | **End** | **Score** | **Expect** | **Sequence** | **Modification** |
| --- | --- | --- | --- | --- | --- | --- | --- | --- | --- |
| 2A5G_HUMAN | Serine/threonine-protein phosphatase 2A 56 kDa regulatory subunit gamma isoform | 763.38 | 2 | 305 | 317 | 51 | 4.5.E-02 | LNELEEILDVIEP |  |
| A1AT_HUMAN | Alpha-1-antitrypsin | 457.26 | 2 | 387 | 394 | 53 | 1.8.E-02 | KVVNPTQK |  |
| A1AT_HUMAN | Alpha-1-antitrypsin | 485.78 | 2 | 386 | 394 | 56 | 8.0.E-03 | GKVVNPTQK |  |
| A1AT_HUMAN | Alpha-1-antitrypsin | 551.29 | 2 | 385 | 394 | 55 | 1.7.E-02 | MGKVVNPTQK |  |
| A1AT_HUMAN | Alpha-1-antitrypsin | 559.30 | 2 | 385 | 394 | 76 | 1.2.E-04 | MGKVVNPTQK | Oxidation (M) |
| A1AT_HUMAN | Alpha-1-antitrypsin | 624.82 | 2 | 384 | 394 | 63 | 2.7.E-03 | FMGKVVNPTQK |  |
| A1AT_HUMAN | Alpha-1-antitrypsin | 637.76 | 2 | 1 | 12 | 50 | 1.6.E-02 | EDPQGDAAQKTD |  |
| A1AT_HUMAN | Alpha-1-antitrypsin | 688.28 | 2 | 1 | 13 | 45 | 5.5.E-02 | EDPQGDAAQKTDT |  |
| A1AT_HUMAN | Alpha-1-antitrypsin | 709.38 | 2 | 381 | 393 | 55 | 1.7.E-02 | SPLFMGKVVNPTQ |  |
| A1AT_HUMAN | Alpha-1-antitrypsin | 731.81 | 2 | 1 | 14 | 50 | 2.7.E-02 | EDPQGDAAQKTDTS |  |
| A1AT_HUMAN | Alpha-1-antitrypsin | 533.89 | 3 | 1 | 15 | 56 | 4.9.E-03 | EDPQGDAAQKTDTSH |  |
| A1AT_HUMAN | Alpha-1-antitrypsin | 569.24 | 3 | 6 | 20 | 50 | 2.8.E-02 | DAAQKTDTSHHDQDH |  |
| A1AT_HUMAN | Alpha-1-antitrypsin | 592.32 | 3 | 379 | 394 | 55 | 1.7.E-02 | TKSPLFMGKVVNPTQK |  |
| A1AT_HUMAN | Alpha-1-antitrypsin | 597.65 | 3 | 379 | 394 | 58 | 9.4.E-03 | TKSPLFMGKVVNPTQK | Oxidation (M) |
| A1AT_HUMAN | Alpha-1-antitrypsin | 627.67 | 3 | 332 | 350 | 56 | 1.3.E-02 | AVHKAVLTIDEKGTEAAGA |  |
| A1AT_HUMAN | Alpha-1-antitrypsin | 660.60 | 3 | 1 | 18 | 44 | 5.8.E-02 | EDPQGDAAQKTDTSHHDQ |  |
| A1AT_HUMAN | Alpha-1-antitrypsin | 671.35 | 3 | 332 | 351 | 58 | 9.0.E-03 | AVHKAVLTIDEKGTEAAGAM |  |
| A1AT_HUMAN | Alpha-1-antitrypsin | 676.68 | 3 | 332 | 351 | 50 | 5.9.E-02 | AVHKAVLTIDEKGTEAAGAM | Oxidation (M) |
| A1AT_HUMAN | Alpha-1-antitrypsin | 725.71 | 3 | 332 | 352 | 53 | 3.1.E-02 | AVHKAVLTIDEKGTEAAGAMF | Oxidation (M) |
| A1AT_HUMAN | Alpha-1-antitrypsin | 744.62 | 3 | 1 | 20 | 51 | 7.4.E-03 | EDPQGDAAQKTDTSHHDQDH |  |
| A1AT_HUMAN | Alpha-1-antitrypsin | 781.17 | 4 | 368 | 394 | 66 | 1.7.E-03 | KPFVFLMIEQNTKSPLFMGKVVNPTQK |  |
| A1AT_HUMAN | Alpha-1-antitrypsin | 521.27 | 3 | 381 | 394 | 56 | 1.3.E-02 | SPLFMGKVVNPTQK | Oxidation (M) |
| A1AT_HUMAN | Alpha-1-antitrypsin | 580.25 | 2 | 1 | 11 | 49 | 3.7.E-02 | EDPQGDAAQKT |  |
| A1AT_HUMAN | Alpha-1-antitrypsin | 515.94 | 3 | 381 | 394 | 55 | 1.6.E-02 | SPLFMGKVVNPTQK |  |
| A1AT_HUMAN | Alpha-1-antitrypsin | 617.92 | 3 | 1 | 17 | 50 | 1.7.E-02 | EDPQGDAAQKTDTSHHD |  |
| A1AT_HUMAN | Alpha-1-antitrypsin | 630.33 | 3 | 378 | 394 | 50 | 5.5.E-02 | NTKSPLFMGKVVNPTQK |  |
| A1BG_HUMAN | Alpha-1B-glycoprotein | 478.75 | 2 | 466 | 474 | 54 | 1.5.E-02 | PVELLVAES |  |
| A2MG_HUMAN | Alpha-2-macroglobulin | 478.70 | 2 | 685 | 692 | 47 | 3.9.E-02 | YESDVMGR |  |
| A2MG_HUMAN | Alpha-2-macroglobulin | 630.28 | 2 | 682 | 692 | 58 | 5.0.E-03 | VGFYESDVMGR |  |
| A2MG_HUMAN | Alpha-2-macroglobulin | 638.28 | 2 | 682 | 692 | 58 | 5.4.E-03 | VGFYESDVMGR | Oxidation (M) |
| AACT_HUMAN | Alpha-1-antichymotrypsin | 576.26 | 2 | 1 | 10 | 51 | 3.0.E-02 | HPNSPLDEEN |  |
| AACT_HUMAN | Alpha-1-antichymotrypsin | 868.87 | 2 | 1 | 15 | 52 | 1.6.E-02 | HPNSPLDEENLTQEN |  |
| AACT_HUMAN | Alpha-1-antichymotrypsin | 932.91 | 2 | 1 | 16 | 50 | 3.0.E-02 | HPNSPLDEENLTQENQ |  |
| AACT_HUMAN | Alpha-1-antichymotrypsin | 712.64 | 3 | 1 | 18 | 63 | 1.9.E-03 | HPNSPLDEENLTQENQDR |  |
| AACT_HUMAN | Alpha-1-antichymotrypsin | 765.34 | 3 | 1 | 20 | 53 | 1.9.E-02 | HPNSPLDEENLTQENQDRGT |  |
| ACCN4_HUMAN | Amiloride-sensitive cation channel 4 | 551.75 | 2 | 613 | 624 | 52 | 2.3.E-02 | CPSLGRAEGGGV |  |
| ACTB_HUMAN | Actin, cytoplasmic 1 | 652.01 | 3 | 96 | 113 | 53 | 2.8.E-02 | VAPEEHPVLLTEAPLNPK |  |
| ACTBL_HUMAN | Beta-actin-like protein 2 | 652.04 | 3 | 97 | 114 | 48 | 5.4.E-02 | VAPDEHPILLTEAPLNPK |  |
| AKT2_HUMAN | RAC-beta serine/threonine-protein kinase | 448.73 | 2 | 47 | 54 | 49 | 5.2.E-02 | QTLPPLNN |  |
| ALBU_HUMAN | Serum albumin | 450.75 | 2 | 582 | 591 | 51 | 3.2.E-02 | VAASQAALGL |  |
| ALBU_HUMAN | Serum albumin | 507.29 | 2 | 581 | 591 | 58 | 5.7.E-03 | LVAASQAALGL |  |
| ALBU_HUMAN | Serum albumin | 514.81 | 2 | 580 | 590 | 49 | 3.7.E-02 | KLVAASQAALG |  |
| ALBU_HUMAN | Serum albumin | 571.33 | 2 | 580 | 591 | 59 | 4.4.E-03 | KLVAASQAALGL |  |
| ALBU_HUMAN | Serum albumin | 575.27 | 2 | 7 | 16 | 61 | 3.3.E-03 | DAHKSEVAHR |  |
| ALBU_HUMAN | Serum albumin | 578.83 | 2 | 579 | 590 | 53 | 1.5.E-02 | KKLVAASQAALG |  |
| ALBU_HUMAN | Serum albumin | 607.36 | 2 | 578 | 590 | 48 | 3.9.E-02 | GKKLVAASQAALG |  |
| ALBU_HUMAN | Serum albumin | 635.38 | 2 | 579 | 591 | 60 | 2.4.E-03 | KKLVAASQAALGL |  |
| ALBU_HUMAN | Serum albumin | 547.31 | 3 | 420 | 434 | 65 | 1.3.E-03 | KVPQVSTPTLVEVSR |  |
| ALBU_HUMAN | Serum albumin | 601.66 | 3 | 574 | 591 | 52 | 3.8.E-02 | FAEEGKKLVAASQAALGL |  |
| ALBU_HUMAN | Serum albumin | 475.58 | 3 | 7 | 18 | 53 | 2.3.E-02 | DAHKSEVAHRFK |  |
| AMBP_HUMAN | Protein AMBP | 590.28 | 2 | 324 | 333 | 53 | 2.0.E-02 | GDEELLRFSN |  |
| AMBP_HUMAN | Protein AMBP | 802.88 | 2 | 319 | 333 | 50 | 4.9.E-02 | GVPGDGDEELLRFSN |  |
| ANXA1_HUMAN | Annexin A1 | 870.37 | 2 | 189 | 204 | 110 | 2.6.E-08 | SEDFGVNEDLADSDAR |  |
| APOA1_HUMAN | Apolipoprotein A-I | 508.93 | 3 | 237 | 249 | 61 | 4.2.E-03 | SALEEYTKKLNTQ |  |
| APOA1_HUMAN | Apolipoprotein A-I | 657.68 | 3 | 233 | 249 | 62 | 3.6.E-03 | VSFLSALEEYTKKLNTQ |  |
| APOA1_HUMAN | Apolipoprotein A-I | 685.03 | 3 | 202 | 220 | 52 | 3.2.E-02 | ATEHLSTLSEKAKPALEDL |  |
| APOA1_HUMAN | Apolipoprotein A-I | 784.41 | 4 | 130 | 156 | 52 | 4.0.E-02 | AELQEGARQKLHELQEKLSPLGEEMRD |  |
| APOA1_HUMAN | Apolipoprotein A-I | 841.19 | 4 | 130 | 158 | 61 | 6.1.E-03 | AELQEGARQKLHELQEKLSPLGEEMRDRA |  |
| APOA1_HUMAN | Apolipoprotein A-I | 627.34 | 2 | 240 | 249 | 49 | 5.3.E-02 | EEYTKKLNTQ |  |
| APOA1_HUMAN | Apolipoprotein A-I | 615.00 | 3 | 233 | 248 | 52 | 3.9.E-02 | VSFLSALEEYTKKLNT |  |
| APOA4_HUMAN | Apolipoprotein A-IV | 572.77 | 2 | 275 | 283 | 56 | 9.2.E-03 | HLDQQVEEF |  |
| APOA4_HUMAN | Apolipoprotein A-IV | 629.77 | 2 | 273 | 283 | 51 | 1.8.E-02 | GGHLDQQVEEF |  |
| APOA4_HUMAN | Apolipoprotein A-IV | 750.85 | 2 | 271 | 283 | 64 | 1.8.E-03 | ELGGHLDQQVEEF |  |
| APOA4_HUMAN | Apolipoprotein A-IV | 786.35 | 2 | 270 | 283 | 70 | 3.1.E-04 | AELGGHLDQQVEEF |  |
| APOA4_HUMAN | Apolipoprotein A-IV | 789.39 | 2 | 202 | 215 | 61 | 3.9.E-03 | SLAPYAQDTQEKLN |  |
| APOA4_HUMAN | Apolipoprotein A-IV | 812.89 | 2 | 268 | 282 | 54 | 1.8.E-02 | SLAELGGHLDQQVEE |  |
| APOA4_HUMAN | Apolipoprotein A-IV | 842.91 | 2 | 269 | 283 | 58 | 9.3.E-03 | LAELGGHLDQQVEEF |  |
| APOA4_HUMAN | Apolipoprotein A-IV | 886.42 | 2 | 268 | 283 | 66 | 1.4.E-03 | SLAELGGHLDQQVEEF |  |
| APOA4_HUMAN | Apolipoprotein A-IV | 610.66 | 3 | 230 | 245 | 51 | 4.7.E-02 | EELKARISASAEELRQ |  |
| APOA4_HUMAN | Apolipoprotein A-IV | 613.65 | 3 | 260 | 277 | 54 | 2.0.E-02 | GNTEGLQKSLAELGGHLD |  |
| APOA4_HUMAN | Apolipoprotein A-IV | 614.97 | 3 | 202 | 217 | 61 | 4.6.E-03 | SLAPYAQDTQEKLNHQ |  |
| APOA4_HUMAN | Apolipoprotein A-IV | 634.32 | 3 | 229 | 245 | 59 | 7.5.E-03 | AEELKARISASAEELRQ |  |
| APOA4_HUMAN | Apolipoprotein A-IV | 643.32 | 3 | 268 | 284 | 58 | 8.9.E-03 | SLAELGGHLDQQVEEFR |  |
| APOA4_HUMAN | Apolipoprotein A-IV | 672.34 | 3 | 228 | 245 | 64 | 2.3.E-03 | NAEELKARISASAEELRQ |  |
| APOA4_HUMAN | Apolipoprotein A-IV | 633.34 | 4 | 224 | 245 | 67 | 1.4.E-03 | QMKKNAEELKARISASAEELRQ |  |
| APOA4_HUMAN | Apolipoprotein A-IV | 867.10 | 3 | 260 | 283 | 53 | 3.2.E-02 | GNTEGLQKSLAELGGHLDQQVEEF |  |
| APOA4_HUMAN | Apolipoprotein A-IV | 689.60 | 4 | 260 | 284 | 78 | 1.2.E-04 | GNTEGLQKSLAELGGHLDQQVEEFR |  |
| APOA4_HUMAN | Apolipoprotein A-IV | 634.35 | 2 | 247 | 258 | 57 | 7.8.E-03 | LAPLAEDVRGNL |  |
| APOA4_HUMAN | Apolipoprotein A-IV | 728.62 | 4 | 260 | 285 | 63 | 3.1.E-03 | GNTEGLQKSLAELGGHLDQQVEEFRR |  |
| APOC1_HUMAN | Apolipoprotein C-I | 574.28 | 3 | 1 | 16 | 67 | 1.2.E-03 | TPDVSSALDKLKEFGN |  |
| APOC2_HUMAN | Apolipoprotein C-II | 659.35 | 2 | 68 | 79 | 53 | 2.4.E-02 | TDQVLSVLKGEE |  |
| APOC3_HUMAN | Apolipoprotein C-III | 844.92 | 2 | 24 | 39 | 85 | 1.8.E-05 | KDALSSVQESQVAQQA |  |
| APOC3_HUMAN | Apolipoprotein C-III | 620.98 | 3 | 22 | 39 | 60 | 5.8.E-03 | TAKDALSSVQESQVAQQA |  |
| APOC3_HUMAN | Apolipoprotein C-III | 766.73 | 3 | 18 | 39 | 54 | 2.2.E-02 | HATKTAKDALSSVQESQVAQQA |  |
| APOE_HUMAN | Apolipoprotein E | 663.36 | 2 | 195 | 207 | 60 | 4.5.E-03 | VGSLAGQPLQERA |  |
| APOE_HUMAN | Apolipoprotein E | 678.36 | 2 | 194 | 206 | 51 | 3.5.E-02 | TVGSLAGQPLQER |  |
| APOE_HUMAN | Apolipoprotein E | 713.86 | 2 | 194 | 207 | 59 | 5.9.E-03 | TVGSLAGQPLQERA |  |
| APOE_HUMAN | Apolipoprotein E | 799.37 | 2 | 1 | 14 | 67 | 9.8.E-04 | KVEQAVETEPEPEL |  |
| APOE_HUMAN | Apolipoprotein E | 813.41 | 2 | 194 | 209 | 54 | 2.0.E-02 | TVGSLAGQPLQERAQA |  |
| APOE_HUMAN | Apolipoprotein E | 999.51 | 2 | 194 | 212 | 68 | 9.9.E-04 | TVGSLAGQPLQERAQAWGE |  |
| APOE_HUMAN | Apolipoprotein E | 670.66 | 3 | 1 | 17 | 52 | 3.2.E-02 | KVEQAVETEPEPELRQQ |  |
| APOE_HUMAN | Apolipoprotein E | 756.38 | 3 | 194 | 214 | 68 | 9.6.E-04 | TVGSLAGQPLQERAQAWGERL |  |
| APOE_HUMAN | Apolipoprotein E | 803.76 | 3 | 192 | 214 | 75 | 1.8.E-04 | AATVGSLAGQPLQERAQAWGERL |  |
| APOF_HUMAN | Apolipoprotein F | 668.81 | 2 | 278 | 290 | 57 | 9.1.E-03 | SYDLDPGAGSLEI |  |
| APOF_HUMAN | Apolipoprotein F | 543.76 | 2 | 280 | 290 | 50 | 4.4.E-02 | DLDPGAGSLEI |  |
| APOL1_HUMAN | Apolipoprotein L1 | 766.36 | 2 | 280 | 293 | 55 | 1.4.E-02 | TEPISAESGEQVER |  |
| APOL1_HUMAN | Apolipoprotein L1 | 880.07 | 3 | 1 | 26 | 59 | 7.2.E-03 | EEAGARVQQNVPSGTDTGDPQSKPLG |  |
| BEND7_HUMAN | BEN domain-containing protein 7 | 796.93 | 2 | 230 | 243 | 59 | 6.6.E-03 | TVKQKPSGSEMEKK | Oxidation (M) |
| CALD1_HUMAN | Caldesmon | 646.73 | 2 | 30 | 40 | 39 | 3.8.E-02 | NDDDEEEAARE |  |
| CB027_HUMAN | Uncharacterized protein C2orf27 | 438.20 | 2 | 39 | 46 | 54 | 1.6.E-02 | TALELEEA |  |
| CCD11_HUMAN | Coiled-coil domain-containing protein 11 | 459.23 | 2 | 255 | 262 | 54 | 2.0.E-02 | EARLVESN |  |
| CD181_HUMAN | Putative uncharacterized protein C14orf181 | 456.72 | 2 | 127 | 137 | 55 | 1.1.E-02 | GEGGSAPALGP |  |
| CFAB_HUMAN | Complement factor B | 556.79 | 2 | 1 | 10 | 51 | 3.7.E-02 | TPWSLARPQG |  |
| CO3_HUMAN | Complement C3 | 528.26 | 2 | 1289 | 1297 | 63 | 2.1.E-03 | IHWESASLL |  |
| CO3_HUMAN | Complement C3 | 564.34 | 2 | 1272 | 1281 | 53 | 1.5.E-02 | LDVSLQLPSR |  |
| CO3_HUMAN | Complement C3 | 404.55 | 3 | 1289 | 1298 | 50 | 4.5.E-02 | IHWESASLLR |  |
| CO3_HUMAN | Complement C3 | 456.58 | 3 | 1288 | 1298 | 62 | 3.0.E-03 | RIHWESASLLR |  |
| CO3_HUMAN | Complement C3 | 691.82 | 2 | 1302 | 1314 | 67 | 7.6.E-04 | TKENEGFTVTAEG |  |
| CO3_HUMAN | Complement C3 | 820.86 | 2 | 1300 | 1314 | 54 | 1.6.E-02 | EETKENEGFTVTAEG |  |
| CO3_HUMAN | Complement C3 | 864.35 | 2 | 1299 | 1314 | 96 | 4.9.E-07 | SEETKENEGFTVTAEG |  |
| CO3_HUMAN | Complement C3 | 590.26 | 3 | 1300 | 1315 | 58 | 5.4.E-03 | EETKENEGFTVTAEGK |  |
| CO3_HUMAN | Complement C3 | 619.27 | 3 | 1299 | 1315 | 75 | 8.9.E-05 | SEETKENEGFTVTAEGK |  |
| CO3_HUMAN | Complement C3 | 471.73 | 2 | 1290 | 1297 | 49 | 4.4.E-02 | HWESASLL |  |
| CO3_HUMAN | Complement C3 | 484.28 | 4 | 1283 | 1298 | 52 | 2.6.E-02 | SKITHRIHWESASLLR |  |
| CO3_HUMAN | Complement C3 | 506.03 | 4 | 1282 | 1298 | 60 | 5.1.E-03 | SSKITHRIHWESASLLR |  |
| CO3_HUMAN | Complement C3 | 402.71 | 2 | 650 | 656 | 57 | 1.1.E-02 | SVQLTEK |  |
| CO4A_HUMAN | Complement C4-A | 504.93 | 3 | 1319 | 1331 | 61 | 3.7.E-03 | GFKSHALQLNNRQ |  |
| CO4A_HUMAN | Complement C4-A | 542.63 | 3 | 1319 | 1332 | 52 | 3.4.E-02 | GFKSHALQLNNRQI |  |
| CO4A_HUMAN | Complement C4-A | 542.95 | 3 | 1318 | 1331 | 60 | 4.9.E-03 | NGFKSHALQLNNRQ |  |
| CO4A_HUMAN | Complement C4-A | 405.20 | 2 | 1323 | 1329 | 54 | 1.7.E-02 | HALQLNN |  |
| CO4A_HUMAN | Complement C4-A | 448.72 | 2 | 1322 | 1329 | 52 | 2.2.E-02 | SHALQLNN |  |
| CO4A_HUMAN | Complement C4-A | 569.76 | 2 | 1336 | 1345 | 53 | 2.0.E-02 | EEELQFSLGS |  |
| CO4A_HUMAN | Complement C4-A | 500.27 | 3 | 1318 | 1330 | 61 | 4.1.E-03 | NGFKSHALQLNNR |  |
| CO4B_HUMAN | Complement C4-B | 526.24 | 2 | 1334 | 1342 | 52 | 2.3.E-02 | GLEEELQFS |  |
| CO4B_HUMAN | Complement C4-B | 718.84 | 2 | 1334 | 1346 | 88 | 7.6.E-06 | GLEEELQFSLGSK |  |
| CO4B_HUMAN | Complement C4-B | 747.35 | 2 | 1336 | 1348 | 52 | 2.9.E-02 | EEELQFSLGSKIN |  |
| CO4B_HUMAN | Complement C4-B | 796.89 | 2 | 1336 | 1349 | 55 | 1.8.E-02 | EEELQFSLGSKINV |  |
| CO4B_HUMAN | Complement C4-B | 832.40 | 2 | 1334 | 1348 | 62 | 3.2.E-03 | GLEEELQFSLGSKIN |  |
| CO4B_HUMAN | Complement C4-B | 580.64 | 3 | 1318 | 1332 | 51 | 3.9.E-02 | NGFKSHALQLNNRQI |  |
| CO4B_HUMAN | Complement C4-B | 881.97 | 2 | 1334 | 1349 | 66 | 1.2.E-03 | GLEEELQFSLGSKINV |  |
| CO4B_HUMAN | Complement C4-B | 626.67 | 3 | 1412 | 1428 | 53 | 3.0.E-02 | PDAPLQPVTPLQLFEGR |  |
| CO4B_HUMAN | Complement C4-B | 630.99 | 3 | 1334 | 1350 | 57 | 1.2.E-02 | GLEEELQFSLGSKINVK |  |
| CO4B_HUMAN | Complement C4-B | 703.36 | 3 | 1410 | 1428 | 56 | 1.5.E-02 | DDPDAPLQPVTPLQLFEGR |  |
| CO4B_HUMAN | Complement C4-B | 769.05 | 3 | 1334 | 1355 | 65 | 1.8.E-03 | GLEEELQFSLGSKINVKVGGNS |  |
| CRNN_HUMAN | Cornulin | 714.09 | 4 | 336 | 362 | 55 | 2.1.E-02 | SQTSQAVTGGHTQIQAGSHTETVEQDR |  |
| CUL9_HUMAN | Cullin-9 | 421.20 | 2 | 9 | 16 | 52 | 1.8.E-02 | DLMVPLGP |  |
| CYTSA_HUMAN | Cytospin-A | 537.26 | 2 | 249 | 257 | 55 | 1.2.E-02 | QNTAIREEL |  |
| DERPC_HUMAN | Protein DERPC | 456.72 | 2 | 495 | 504 | 52 | 2.4.E-02 | VGSLPGTNPA |  |
| ECM1_HUMAN | Extracellular matrix protein 1 | 512.24 | 2 | 3 | 12 | 52 | 2.5.E-02 | EGGFTATGQR |  |
| ENK15_HUMAN | HERV-K_3q21.2 provirus ancestral Env polyprotein | 531.28 | 2 | 53 | 61 | 55 | 1.7.E-02 | CLMPAVQNW |  |
| ERBB2_HUMAN | Receptor tyrosine-protein kinase erbB-2 | 733.34 | 2 | 352 | 365 | 54 | 1.8.E-02 | LAFLPESFDGDPAS |  |
| F13A_HUMAN | Coagulation factor XIII A chain | 711.90 | 2 | 26 | 38 | 66 | 1.1.E-03 | DLPTVELQGVVPR |  |
| F13A_HUMAN | Coagulation factor XIII A chain | 744.81 | 2 | 18 | 31 | 57 | 4.5.E-03 | NNSNAAEDDLPTVE |  |
| F13A_HUMAN | Coagulation factor XIII A chain | 769.41 | 2 | 25 | 38 | 52 | 2.9.E-02 | DDLPTVELQGVVPR |  |
| F13A_HUMAN | Coagulation factor XIII A chain | 833.93 | 2 | 24 | 38 | 60 | 5.7.E-03 | EDDLPTVELQGVVPR |  |
| F13A_HUMAN | Coagulation factor XIII A chain | 869.45 | 2 | 23 | 38 | 62 | 3.5.E-03 | AEDDLPTVELQGVVPR |  |
| F13A_HUMAN | Coagulation factor XIII A chain | 641.65 | 3 | 21 | 38 | 69 | 6.7.E-04 | NAAEDDLPTVELQGVVPR |  |
| F13A_HUMAN | Coagulation factor XIII A chain | 746.68 | 3 | 18 | 38 | 83 | 2.7.E-05 | NNSNAAEDDLPTVELQGVVPR |  |
| F13A_HUMAN | Coagulation factor XIII A chain | 844.41 | 3 | 15 | 38 | 74 | 2.6.E-04 | VPPNNSNAAEDDLPTVELQGVVPR |  |
| F13A_HUMAN | Coagulation factor XIII A chain | 868.08 | 3 | 14 | 38 | 58 | 9.7.E-03 | AVPPNNSNAAEDDLPTVELQGVVPR |  |
| F13A_HUMAN | Coagulation factor XIII A chain | 920.12 | 3 | 13 | 38 | 97 | 1.4.E-06 | RAVPPNNSNAAEDDLPTVELQGVVPR |  |
| F13A_HUMAN | Coagulation factor XIII A chain | 837.66 | 4 | 7 | 38 | 71 | 5.2.E-04 | TAFGGRRAVPPNNSNAAEDDLPTVELQGVVPR |  |
| FETUA_HUMAN | Alpha-2-HS-glycoprotein | 500.27 | 2 | 323 | 333 | 48 | 6.1.E-02 | TVVQPSVGAAA |  |
| FETUA_HUMAN | Alpha-2-HS-glycoprotein | 549.28 | 2 | 312 | 321 | 51 | 3.5.E-02 | SGEVSHPRKT |  |
| FETUA_HUMAN | Alpha-2-HS-glycoprotein | 578.30 | 4 | 300 | 321 | 68 | 9.3.E-04 | HTFMGVVSLGSPSGEVSHPRKT |  |
| FETUA_HUMAN | Alpha-2-HS-glycoprotein | 582.30 | 4 | 300 | 321 | 52 | 3.9.E-02 | HTFMGVVSLGSPSGEVSHPRKT | Oxidation (M) |
| FETUA_HUMAN | Alpha-2-HS-glycoprotein | 526.76 | 2 | 310 | 319 | 51 | 3.2.E-02 | SPSGEVSHPR |  |
| FETUA_HUMAN | Alpha-2-HS-glycoprotein | 528.78 | 2 | 323 | 334 | 54 | 1.7.E-02 | TVVQPSVGAAAG |  |
| FETUA_HUMAN | Alpha-2-HS-glycoprotein | 598.65 | 3 | 304 | 321 | 54 | 2.2.E-02 | GVVSLGSPSGEVSHPRKT |  |
| FIBA_HUMAN | Fibrinogen alpha chain | 432.69 | 2 | 7 | 15 | 52 | 1.8.E-02 | DFLAEGGGV |  |
| FIBA_HUMAN | Fibrinogen alpha chain | 479.21 | 2 | 573 | 581 | 53 | 1.2.E-02 | GDSTFESKS |  |
| FIBA_HUMAN | Fibrinogen alpha chain | 510.74 | 2 | 7 | 16 | 50 | 4.2.E-02 | DFLAEGGGVR |  |
| FIBA_HUMAN | Fibrinogen alpha chain | 525.73 | 2 | 5 | 15 | 48 | 4.2.E-02 | EGDFLAEGGGV |  |
| FIBA_HUMAN | Fibrinogen alpha chain | 539.25 | 2 | 6 | 16 | 62 | 2.7.E-03 | GDFLAEGGGVR |  |
| FIBA_HUMAN | Fibrinogen alpha chain | 544.23 | 2 | 565 | 574 | 53 | 1.1.E-02 | TSSTSYNRGD |  |
| FIBA_HUMAN | Fibrinogen alpha chain | 554.24 | 2 | 4 | 15 | 60 | 2.6.E-03 | GEGDFLAEGGGV |  |
| FIBA_HUMAN | Fibrinogen alpha chain | 560.73 | 2 | 573 | 582 | 53 | 1.2.E-02 | GDSTFESKSY |  |
| FIBA_HUMAN | Fibrinogen alpha chain | 597.75 | 2 | 3 | 15 | 51 | 1.7.E-02 | SGEGDFLAEGGGV |  |
| FIBA_HUMAN | Fibrinogen alpha chain | 603.77 | 2 | 5 | 16 | 53 | 1.9.E-02 | EGDFLAEGGGVR |  |
| FIBA_HUMAN | Fibrinogen alpha chain | 609.23 | 2 | 586 | 597 | 55 | 2.3.E-03 | DEAGSEADHEGT |  |
| FIBA_HUMAN | Fibrinogen alpha chain | 614.24 | 2 | 589 | 600 | 65 | 4.1.E-04 | GSEADHEGTHST |  |
| FIBA_HUMAN | Fibrinogen alpha chain | 632.28 | 2 | 4 | 16 | 52 | 2.4.E-02 | GEGDFLAEGGGVR |  |
| FIBA_HUMAN | Fibrinogen alpha chain | 649.75 | 2 | 588 | 600 | 64 | 4.1.E-04 | AGSEADHEGTHST |  |
| FIBA_HUMAN | Fibrinogen alpha chain | 655.26 | 2 | 2 | 15 | 50 | 1.6.E-02 | DSGEGDFLAEGGGV |  |
| FIBA_HUMAN | Fibrinogen alpha chain | 675.79 | 2 | 3 | 16 | 73 | 1.5.E-04 | SGEGDFLAEGGGVR |  |
| FIBA_HUMAN | Fibrinogen alpha chain | 452.17 | 3 | 586 | 598 | 55 | 2.7.E-03 | DEAGSEADHEGTH |  |
| FIBA_HUMAN | Fibrinogen alpha chain | 690.78 | 2 | 1 | 15 | 56 | 4.0.E-03 | ADSGEGDFLAEGGGV |  |
| FIBA_HUMAN | Fibrinogen alpha chain | 699.35 | 2 | 425 | 438 | 49 | 6.0.E-02 | TGKEKVTSGSTTTT |  |
| FIBA_HUMAN | Fibrinogen alpha chain | 711.81 | 2 | 564 | 576 | 52 | 1.9.E-02 | FTSSTSYNRGDST |  |
| FIBA_HUMAN | Fibrinogen alpha chain | 714.29 | 2 | 587 | 600 | 50 | 1.5.E-02 | EAGSEADHEGTHST |  |
| FIBA_HUMAN | Fibrinogen alpha chain | 733.31 | 2 | 2 | 16 | 70 | 2.8.E-04 | DSGEGDFLAEGGGVR |  |
| FIBA_HUMAN | Fibrinogen alpha chain | 750.34 | 2 | 559 | 571 | 63 | 1.9.E-03 | SYSKQFTSSTSYN |  |
| FIBA_HUMAN | Fibrinogen alpha chain | 768.83 | 2 | 1 | 16 | 104 | 1.1.E-07 | ADSGEGDFLAEGGGVR |  |
| FIBA_HUMAN | Fibrinogen alpha chain | 514.86 | 3 | 586 | 600 | 43 | 2.7.E-02 | DEAGSEADHEGTHST |  |
| FIBA_HUMAN | Fibrinogen alpha chain | 518.60 | 3 | 425 | 439 | 64 | 1.9.E-03 | TGKEKVTSGSTTTTR |  |
| FIBA_HUMAN | Fibrinogen alpha chain | 538.54 | 3 | 585 | 600 | 53 | 3.8.E-03 | ADEAGSEADHEGTHST |  |
| FIBA_HUMAN | Fibrinogen alpha chain | 557.57 | 3 | 586 | 601 | 58 | 4.2.E-03 | DEAGSEADHEGTHSTK |  |
| FIBA_HUMAN | Fibrinogen alpha chain | 582.23 | 3 | 584 | 600 | 75 | 2.7.E-05 | MADEAGSEADHEGTHST |  |
| FIBA_HUMAN | Fibrinogen alpha chain | 587.56 | 3 | 584 | 600 | 68 | 1.5.E-04 | MADEAGSEADHEGTHST | Oxidation (M) |
| FIBA_HUMAN | Fibrinogen alpha chain | 609.60 | 3 | 586 | 602 | 59 | 4.9.E-03 | DEAGSEADHEGTHSTKR |  |
| FIBA_HUMAN | Fibrinogen alpha chain | 616.62 | 3 | 588 | 605 | 52 | 2.5.E-02 | AGSEADHEGTHSTKRGHA |  |
| FIBA_HUMAN | Fibrinogen alpha chain | 624.91 | 3 | 583 | 600 | 61 | 8.1.E-04 | KMADEAGSEADHEGTHST |  |
| FIBA_HUMAN | Fibrinogen alpha chain | 624.93 | 3 | 584 | 601 | 72 | 1.8.E-04 | MADEAGSEADHEGTHSTK |  |
| FIBA_HUMAN | Fibrinogen alpha chain | 628.59 | 3 | 586 | 603 | 46 | 4.7.E-02 | DEAGSEADHEGTHSTKRG |  |
| FIBA_HUMAN | Fibrinogen alpha chain | 630.26 | 3 | 583 | 600 | 59 | 2.0.E-03 | KMADEAGSEADHEGTHST | Oxidation (M) |
| FIBA_HUMAN | Fibrinogen alpha chain | 652.28 | 3 | 585 | 603 | 63 | 1.7.E-03 | ADEAGSEADHEGTHSTKRG |  |
| FIBA_HUMAN | Fibrinogen alpha chain | 667.97 | 3 | 557 | 574 | 71 | 2.9.E-04 | SSSYSKQFTSSTSYNRGD |  |
| FIBA_HUMAN | Fibrinogen alpha chain | 674.28 | 3 | 586 | 604 | 71 | 1.8.E-04 | DEAGSEADHEGTHSTKRGH |  |
| FIBA_HUMAN | Fibrinogen alpha chain | 697.96 | 3 | 586 | 605 | 83 | 1.0.E-05 | DEAGSEADHEGTHSTKRGHA |  |
| FIBA_HUMAN | Fibrinogen alpha chain | 701.65 | 3 | 558 | 576 | 50 | 4.6.E-02 | SSYSKQFTSSTSYNRGDST |  |
| FIBA_HUMAN | Fibrinogen alpha chain | 708.28 | 3 | 581 | 600 | 68 | 1.7.E-04 | SYKMADEAGSEADHEGTHST |  |
| FIBA_HUMAN | Fibrinogen alpha chain | 713.60 | 3 | 581 | 600 | 41 | 4.9.E-02 | SYKMADEAGSEADHEGTHST | Oxidation (M) |
| FIBA_HUMAN | Fibrinogen alpha chain | 721.63 | 3 | 585 | 605 | 88 | 3.5.E-06 | ADEAGSEADHEGTHSTKRGHA |  |
| FIBA_HUMAN | Fibrinogen alpha chain | 730.66 | 3 | 557 | 576 | 91 | 3.2.E-06 | SSSYSKQFTSSTSYNRGDST |  |
| FIBA_HUMAN | Fibrinogen alpha chain | 574.25 | 4 | 584 | 605 | 57 | 5.1.E-03 | MADEAGSEADHEGTHSTKRGHA |  |
| FIBA_HUMAN | Fibrinogen alpha chain | 770.65 | 3 | 584 | 605 | 86 | 4.2.E-06 | MADEAGSEADHEGTHSTKRGHA | Oxidation (M) |
| FIBA_HUMAN | Fibrinogen alpha chain | 602.52 | 4 | 581 | 602 | 73 | 2.1.E-04 | SYKMADEAGSEADHEGTHSTKR |  |
| FIBA_HUMAN | Fibrinogen alpha chain | 606.26 | 4 | 583 | 605 | 77 | 5.9.E-05 | KMADEAGSEADHEGTHSTKRGHA |  |
| FIBA_HUMAN | Fibrinogen alpha chain | 610.27 | 4 | 583 | 605 | 60 | 3.9.E-03 | KMADEAGSEADHEGTHSTKRGHA | Oxidation (M) |
| FIBA_HUMAN | Fibrinogen alpha chain | 616.77 | 4 | 581 | 603 | 53 | 1.7.E-02 | SYKMADEAGSEADHEGTHSTKRG |  |
| FIBA_HUMAN | Fibrinogen alpha chain | 617.51 | 4 | 578 | 600 | 55 | 8.4.E-03 | ESKSYKMADEAGSEADHEGTHST |  |
| FIBA_HUMAN | Fibrinogen alpha chain | 668.78 | 4 | 581 | 605 | 69 | 3.4.E-04 | SYKMADEAGSEADHEGTHSTKRGHA |  |
| FIBA_HUMAN | Fibrinogen alpha chain | 672.78 | 4 | 581 | 605 | 74 | 1.0.E-04 | SYKMADEAGSEADHEGTHSTKRGHA | Oxidation (M) |
| FIBA_HUMAN | Fibrinogen alpha chain | 744.30 | 4 | 573 | 600 | 43 | 5.4.E-02 | GDSTFESKSYKMADEAGSEADHEGTHST |  |
| FIBA_HUMAN | Fibrinogen alpha chain | 748.10 | 4 | 583 | 610 | 62 | 3.5.E-03 | KMADEAGSEADHEGTHSTKRGHAKSRPV |  |
| FIBA_HUMAN | Fibrinogen alpha chain | 748.30 | 4 | 573 | 600 | 78 | 1.5.E-05 | GDSTFESKSYKMADEAGSEADHEGTHST | Oxidation (M) |
| FIBA_HUMAN | Fibrinogen alpha chain | 810.62 | 4 | 581 | 610 | 57 | 1.0.E-02 | SYKMADEAGSEADHEGTHSTKRGHAKSRPV |  |
| FIBA_HUMAN | Fibrinogen alpha chain | 811.90 | 4 | 571 | 600 | 67 | 1.4.E-03 | NRGDSTFESKSYKMADEAGSEADHEGTHST |  |
| FIBA_HUMAN | Fibrinogen alpha chain | 814.61 | 4 | 581 | 610 | 49 | 5.2.E-02 | SYKMADEAGSEADHEGTHSTKRGHAKSRPV | Oxidation (M) |
| FIBA_HUMAN | Fibrinogen alpha chain | 877.17 | 4 | 523 | 555 | 65 | 2.3.E-03 | SETESRGSESGIFTNTKESSSHHPGIAEFPSRG |  |
| FIBA_HUMAN | Fibrinogen alpha chain | 435.69 | 2 | 573 | 580 | 50 | 2.2.E-02 | GDSTFESK |  |
| FIBA_HUMAN | Fibrinogen alpha chain | 453.25 | 2 | 8 | 16 | 55 | 1.3.E-02 | FLAEGGGVR |  |
| FIBA_HUMAN | Fibrinogen alpha chain | 461.21 | 2 | 6 | 15 | 53 | 1.9.E-02 | GDFLAEGGGV |  |
| FIBA_HUMAN | Fibrinogen alpha chain | 532.24 | 2 | 574 | 582 | 49 | 4.4.E-02 | DSTFESKSY |  |
| FIBA_HUMAN | Fibrinogen alpha chain | 561.25 | 2 | 557 | 566 | 49 | 4.6.E-02 | SSSYSKQFTS |  |
| FIBA_HUMAN | Fibrinogen alpha chain | 638.28 | 2 | 565 | 576 | 68 | 4.4.E-04 | TSSTSYNRGDST |  |
| FIBA_HUMAN | Fibrinogen alpha chain | 680.32 | 2 | 496 | 508 | 68 | 7.1.E-04 | DEAAFFDTASTGK |  |
| FIBA_HUMAN | Fibrinogen alpha chain | 775.83 | 2 | 563 | 576 | 60 | 2.6.E-03 | QFTSSTSYNRGDST |  |
| FIBA_HUMAN | Fibrinogen alpha chain | 555.91 | 3 | 567 | 581 | 48 | 5.0.E-02 | STSYNRGDSTFESKS |  |
| FIBA_HUMAN | Fibrinogen alpha chain | 574.26 | 3 | 571 | 585 | 69 | 5.0.E-04 | NRGDSTFESKSYKMA |  |
| FIBA_HUMAN | Fibrinogen alpha chain | 893.38 | 2 | 564 | 579 | 48 | 3.8.E-02 | FTSSTSYNRGDSTFES |  |
| FIBA_HUMAN | Fibrinogen alpha chain | 779.69 | 3 | 557 | 577 | 66 | 1.3.E-03 | SSSYSKQFTSSTSYNRGDSTF |  |
| FIBA_HUMAN | Fibrinogen alpha chain | 606.54 | 4 | 564 | 584 | 53 | 2.6.E-02 | FTSSTSYNRGDSTFESKSYKM |  |
| FIBA_HUMAN | Fibrinogen alpha chain | 647.04 | 4 | 582 | 605 | 76 | 1.0.E-04 | YKMADEAGSEADHEGTHSTKRGHA |  |
| FIBA_HUMAN | Fibrinogen alpha chain | 798.36 | 4 | 557 | 584 | 54 | 2.2.E-02 | SSSYSKQFTSSTSYNRGDSTFESKSYKM |  |
| FIBA_HUMAN | Fibrinogen alpha chain | 467.22 | 2 | 557 | 564 | 52 | 2.7.E-02 | SSSYSKQF |  |
| FIBA_HUMAN | Fibrinogen alpha chain | 620.33 | 2 | 427 | 438 | 76 | 1.2.E-04 | KEKVTSGSTTTT |  |
| FIBA_HUMAN | Fibrinogen alpha chain | 625.30 | 2 | 561 | 571 | 50 | 4.2.E-02 | SKQFTSSTSYN |  |
| FIBA_HUMAN | Fibrinogen alpha chain | 464.20 | 3 | 571 | 582 | 52 | 1.7.E-02 | NRGDSTFESKSY |  |
| FIBA_HUMAN | Fibrinogen alpha chain | 737.37 | 2 | 82 | 94 | 61 | 4.2.E-03 | DSHSLTTNIMEIL |  |
| FIBA_HUMAN | Fibrinogen alpha chain | 793.85 | 2 | 558 | 571 | 53 | 1.6.E-02 | SSYSKQFTSSTSYN |  |
| FIBA_HUMAN | Fibrinogen alpha chain | 543.96 | 3 | 82 | 95 | 55 | 1.9.E-02 | DSHSLTTNIMEILR |  |
| FIBA_HUMAN | Fibrinogen alpha chain | 819.86 | 2 | 565 | 579 | 49 | 3.7.E-02 | TSSTSYNRGDSTFES |  |
| FIBA_HUMAN | Fibrinogen alpha chain | 550.60 | 3 | 571 | 584 | 51 | 4.5.E-02 | NRGDSTFESKSYKM |  |
| FIBA_HUMAN | Fibrinogen alpha chain | 555.92 | 3 | 571 | 584 | 54 | 1.6.E-02 | NRGDSTFESKSYKM | Oxidation (M) |
| FIBA_HUMAN | Fibrinogen alpha chain | 837.36 | 2 | 557 | 571 | 53 | 1.4.E-02 | SSSYSKQFTSSTSYN |  |
| FIBA_HUMAN | Fibrinogen alpha chain | 579.60 | 3 | 571 | 585 | 56 | 1.1.E-02 | NRGDSTFESKSYKMA | Oxidation (M) |
| FIBA_HUMAN | Fibrinogen alpha chain | 586.92 | 3 | 492 | 507 | 58 | 4.8.E-03 | HRHPDEAAFFDTASTG |  |
| FIBA_HUMAN | Fibrinogen alpha chain | 592.93 | 3 | 589 | 605 | 52 | 2.1.E-02 | GSEADHEGTHSTKRGHA |  |
| FIBA_HUMAN | Fibrinogen alpha chain | 610.61 | 3 | 557 | 572 | 76 | 1.1.E-04 | SSSYSKQFTSSTSYNR |  |
| FIBA_HUMAN | Fibrinogen alpha chain | 612.60 | 3 | 571 | 586 | 67 | 7.7.E-04 | NRGDSTFESKSYKMAD |  |
| FIBA_HUMAN | Fibrinogen alpha chain | 629.62 | 3 | 492 | 508 | 82 | 2.8.E-05 | HRHPDEAAFFDTASTGK |  |
| FIBA_HUMAN | Fibrinogen alpha chain | 630.26 | 3 | 584 | 601 | 52 | 1.0.E-02 | MADEAGSEADHEGTHSTK | Oxidation (M) |
| FIBA_HUMAN | Fibrinogen alpha chain | 957.41 | 2 | 563 | 579 | 49 | 3.9.E-02 | QFTSSTSYNRGDSTFES |  |
| FIBA_HUMAN | Fibrinogen alpha chain | 645.59 | 3 | 581 | 598 | 61 | 1.1.E-03 | SYKMADEAGSEADHEGTH |  |
| FIBA_HUMAN | Fibrinogen alpha chain | 650.93 | 3 | 581 | 598 | 45 | 4.4.E-02 | SYKMADEAGSEADHEGTH | Oxidation (M) |
| FIBA_HUMAN | Fibrinogen alpha chain | 659.63 | 3 | 587 | 605 | 53 | 2.1.E-02 | EAGSEADHEGTHSTKRGHA |  |
| FIBA_HUMAN | Fibrinogen alpha chain | 674.62 | 3 | 581 | 599 | 51 | 2.3.E-02 | SYKMADEAGSEADHEGTHS |  |
| FIBA_HUMAN | Fibrinogen alpha chain | 676.95 | 3 | 584 | 602 | 81 | 1.6.E-05 | MADEAGSEADHEGTHSTKR |  |
| FIBA_HUMAN | Fibrinogen alpha chain | 679.28 | 3 | 582 | 600 | 51 | 1.3.E-02 | YKMADEAGSEADHEGTHST |  |
| FIBA_HUMAN | Fibrinogen alpha chain | 682.29 | 3 | 584 | 602 | 48 | 3.5.E-02 | MADEAGSEADHEGTHSTKR | Oxidation (M) |
| FIBA_HUMAN | Fibrinogen alpha chain | 563.50 | 4 | 581 | 601 | 50 | 3.1.E-02 | SYKMADEAGSEADHEGTHSTK |  |
| FIBA_HUMAN | Fibrinogen alpha chain | 567.50 | 4 | 581 | 601 | 61 | 2.9.E-03 | SYKMADEAGSEADHEGTHSTK | Oxidation (M) |
| FIBA_HUMAN | Fibrinogen alpha chain | 606.52 | 4 | 581 | 602 | 51 | 3.5.E-02 | SYKMADEAGSEADHEGTHSTKR | Oxidation (M) |
| FIBA_HUMAN | Fibrinogen alpha chain | 620.77 | 4 | 581 | 603 | 54 | 1.5.E-02 | SYKMADEAGSEADHEGTHSTKRG | Oxidation (M) |
| FIBA_HUMAN | Fibrinogen alpha chain | 754.84 | 4 | 578 | 605 | 68 | 7.7.E-04 | ESKSYKMADEAGSEADHEGTHSTKRGHA |  |
| FIBA_HUMAN | Fibrinogen alpha chain | 758.86 | 4 | 578 | 605 | 51 | 4.6.E-02 | ESKSYKMADEAGSEADHEGTHSTKRGHA | Oxidation (M) |
| FIBA_HUMAN | Fibrinogen alpha chain | 815.87 | 4 | 571 | 600 | 71 | 4.0.E-04 | NRGDSTFESKSYKMADEAGSEADHEGTHST | Oxidation (M) |
| FIBA_HUMAN | Fibrinogen alpha chain | 852.64 | 4 | 570 | 600 | 82 | 3.0.E-05 | YNRGDSTFESKSYKMADEAGSEADHEGTHST |  |
| FIBA_HUMAN | Fibrinogen alpha chain | 881.70 | 4 | 573 | 605 | 64 | 3.2.E-03 | GDSTFESKSYKMADEAGSEADHEGTHSTKRGHA |  |
| FIBB_HUMAN | Fibrinogen beta chain | 897.92 | 2 | 24 | 41 | 62 | 3.4.E-03 | EEAPSLRPAPPPISGGGY |  |
| FIBB_HUMAN | Fibrinogen beta chain | 450.68 | 2 | 6 | 13 | 47 | 3.2.E-02 | NEEGFFSA |  |
| FIBB_HUMAN | Fibrinogen beta chain | 565.21 | 2 | 4 | 13 | 42 | 5.8.E-02 | NDNEEGFFSA |  |
| FLNC_HUMAN | Filamin-C | 457.27 | 2 | 325 | 332 | 47 | 5.4.E-02 | KVVPNNDK |  |
| FOSL2_HUMAN | Fos-related antigen 2 | 402.18 | 2 | 291 | 298 | 46 | 5.5.E-02 | ESPASPSE |  |
| GELS_HUMAN | Gelsolin | 563.04 | 4 | 1 | 23 | 56 | 1.5.E-02 | ATASRGASQAGAPQGRVPEARPN |  |
| GELS_HUMAN | Gelsolin | 472.23 | 3 | 2 | 16 | 57 | 1.0.E-02 | TASRGASQAGAPQGR |  |
| GIT1_HUMAN | ARF GTPase-activating protein GIT1 | 403.22 | 2 | 303 | 309 | 53 | 2.7.E-02 | STLVTER |  |
| H2B1C_HUMAN | Histone H2B type 1-C/E/F/G/I | 546.80 | 2 | 2 | 12 | 49 | 5.5.E-02 | PEPAKSAPAPK |  |
| HBB_HUMAN | Hemoglobin subunit beta | 420.22 | 2 | 141 | 147 | 50 | 3.4.E-02 | ALAHKYH |  |
| HPT_HUMAN | Haptoglobin | 405.72 | 2 | 145 | 152 | 49 | 4.1.E-02 | LGGHLDAK |  |
| HPT_HUMAN | Haptoglobin | 668.28 | 2 | 1 | 13 | 48 | 3.7.E-02 | VDSGNDVTDIADD |  |
| HPT_HUMAN | Haptoglobin | 696.79 | 2 | 1 | 14 | 52 | 1.4.E-02 | VDSGNDVTDIADDG |  |
| HPT_HUMAN | Haptoglobin | 610.76 | 2 | 1 | 12 | 59 | 3.0.E-03 | VDSGNDVTDIAD |  |
| HPT_HUMAN | Haptoglobin | 766.41 | 2 | 376 | 388 | 50 | 4.9.E-02 | SIQDWVQKTIAEN |  |
| HPTR_HUMAN | Haptoglobin-related protein | 434.22 | 2 | 86 | 94 | 54 | 1.4.E-02 | LGGHLDAKG |  |
| HPTR_HUMAN | Haptoglobin-related protein | 462.26 | 2 | 85 | 93 | 53 | 1.7.E-02 | ILGGHLDAK |  |
| HPTR_HUMAN | Haptoglobin-related protein | 490.76 | 2 | 85 | 94 | 53 | 1.9.E-02 | ILGGHLDAKG |  |
| HPTR_HUMAN | Haptoglobin-related protein | 546.80 | 2 | 74 | 83 | 53 | 2.3.E-02 | KPKNPANPVQ |  |
| HPTR_HUMAN | Haptoglobin-related protein | 575.32 | 2 | 73 | 83 | 49 | 5.1.E-02 | GKPKNPANPVQ |  |
| HV303_HUMAN | Ig heavy chain V-III region VH26 | 741.89 | 2 | 1 | 15 | 55 | 1.8.E-02 | EVQLLESGGGLVQPG |  |
| HV303_HUMAN | Ig heavy chain V-III region VH26 | 770.40 | 2 | 1 | 16 | 53 | 2.3.E-02 | EVQLLESGGGLVQPGG |  |
| HV305_HUMAN | Ig heavy chain V-III region BRO | 430.72 | 2 | 1 | 8 | 49 | 5.9.E-02 | EVQLVESG |  |
| HV305_HUMAN | Ig heavy chain V-III region BRO | 657.85 | 2 | 1 | 13 | 58 | 8.4.E-03 | EVQLVESGGGLVQ |  |
| HV305_HUMAN | Ig heavy chain V-III region BRO | 734.86 | 2 | 1 | 15 | 53 | 2.7.E-02 | EVQLVESGGGLVQPG |  |
| HV305_HUMAN | Ig heavy chain V-III region BRO | 763.38 | 2 | 1 | 16 | 65 | 1.7.E-03 | EVQLVESGGGLVQPGG |  |
| HV305_HUMAN | Ig heavy chain V-III region BRO | 459.23 | 2 | 1 | 9 | 50 | 4.4.E-02 | EVQLVESGG |  |
| HV307_HUMAN | Ig heavy chain V-III region CAM | 1041.04 | 2 | 1 | 20 | 51 | 5.3.E-02 | QVELVESGGGVVEPGRSLRL |  |
| HV310_HUMAN | Ig heavy chain V-III region HIL | 796.92 | 2 | 1 | 16 | 50 | 4.8.E-02 | QVKLVQAGGGVVQPGR |  |
| IGHG1_HUMAN | Ig gamma-1 chain C region | 590.83 | 2 | 319 | 329 | 53 | 2.0.E-02 | YTQKSLSLSPG |  |
| IGHG1_HUMAN | Ig gamma-1 chain C region | 561.29 | 3 | 315 | 329 | 57 | 1.1.E-02 | LHNHYTQKSLSLSPG |  |
| IGHG2_HUMAN | Ig gamma-2 chain C region | 509.27 | 2 | 316 | 325 | 59 | 6.6.E-03 | TQKSLSLSPG |  |
| IGHG2_HUMAN | Ig gamma-2 chain C region | 584.97 | 3 | 310 | 325 | 53 | 3.0.E-02 | ALHNHYTQKSLSLSPG |  |
| IRK2_HUMAN | Inward rectifier potassium channel 2 | 655.27 | 2 | 383 | 394 | 47 | 4.6.E-02 | TSKEEDDSENGV |  |
| ITIH4_HUMAN | Inter-alpha-trypsin inhibitor heavy chain H4 | 465.70 | 2 | 645 | 653 | 50 | 2.4.E-02 | PGPPDVPDH |  |
| ITIH4_HUMAN | Inter-alpha-trypsin inhibitor heavy chain H4 | 478.77 | 2 | 634 | 642 | 56 | 8.6.E-03 | PGVLSSRQL |  |
| ITIH4_HUMAN | Inter-alpha-trypsin inhibitor heavy chain H4 | 501.23 | 2 | 645 | 654 | 57 | 5.9.E-03 | PGPPDVPDHA |  |
| ITIH4_HUMAN | Inter-alpha-trypsin inhibitor heavy chain H4 | 522.25 | 2 | 644 | 653 | 49 | 4.7.E-02 | LPGPPDVPDH |  |
| ITIH4_HUMAN | Inter-alpha-trypsin inhibitor heavy chain H4 | 550.75 | 2 | 643 | 653 | 52 | 2.2.E-02 | GLPGPPDVPDH |  |
| ITIH4_HUMAN | Inter-alpha-trypsin inhibitor heavy chain H4 | 557.77 | 2 | 644 | 654 | 50 | 4.5.E-02 | LPGPPDVPDHA |  |
| ITIH4_HUMAN | Inter-alpha-trypsin inhibitor heavy chain H4 | 567.79 | 2 | 460 | 470 | 51 | 3.8.E-02 | GSEMVVAGKLQ | Oxidation (M) |
| ITIH4_HUMAN | Inter-alpha-trypsin inhibitor heavy chain H4 | 586.27 | 2 | 643 | 654 | 49 | 4.7.E-02 | GLPGPPDVPDHA |  |
| ITIH4_HUMAN | Inter-alpha-trypsin inhibitor heavy chain H4 | 621.81 | 2 | 643 | 655 | 50 | 4.7.E-02 | GLPGPPDVPDHAA |  |
| ITIH4_HUMAN | Inter-alpha-trypsin inhibitor heavy chain H4 | 703.34 | 2 | 643 | 656 | 67 | 9.9.E-04 | GLPGPPDVPDHAAY |  |
| ITIH4_HUMAN | Inter-alpha-trypsin inhibitor heavy chain H4 | 746.88 | 2 | 635 | 649 | 61 | 4.1.E-03 | GVLSSRQLGLPGPPD |  |
| ITIH4_HUMAN | Inter-alpha-trypsin inhibitor heavy chain H4 | 766.90 | 2 | 445 | 457 | 50 | 5.1.E-02 | AVEEVTQNNFRLL |  |
| ITIH4_HUMAN | Inter-alpha-trypsin inhibitor heavy chain H4 | 795.43 | 2 | 634 | 649 | 51 | 3.9.E-02 | PGVLSSRQLGLPGPPD |  |
| ITIH4_HUMAN | Inter-alpha-trypsin inhibitor heavy chain H4 | 808.85 | 2 | 645 | 659 | 50 | 2.9.E-02 | PGPPDVPDHAAYHPF |  |
| ITIH4_HUMAN | Inter-alpha-trypsin inhibitor heavy chain H4 | 552.62 | 3 | 639 | 654 | 61 | 4.5.E-03 | SRQLGLPGPPDVPDHA |  |
| ITIH4_HUMAN | Inter-alpha-trypsin inhibitor heavy chain H4 | 671.34 | 3 | 635 | 654 | 57 | 1.3.E-02 | GVLSSRQLGLPGPPDVPDHA |  |
| ITIH4_HUMAN | Inter-alpha-trypsin inhibitor heavy chain H4 | 727.39 | 3 | 634 | 655 | 53 | 2.7.E-02 | PGVLSSRQLGLPGPPDVPDHAA |  |
| ITIH4_HUMAN | Inter-alpha-trypsin inhibitor heavy chain H4 | 645.07 | 4 | 634 | 658 | 56 | 1.5.E-02 | PGVLSSRQLGLPGPPDVPDHAAYHP |  |
| ITIH4_HUMAN | Inter-alpha-trypsin inhibitor heavy chain H4 | 681.83 | 4 | 634 | 659 | 51 | 4.8.E-02 | PGVLSSRQLGLPGPPDVPDHAAYHPF |  |
| ITIH4_HUMAN | Inter-alpha-trypsin inhibitor heavy chain H4 | 531.28 | 2 | 461 | 470 | 64 | 1.8.E-03 | SEMVVAGKLQ |  |
| ITIH4_HUMAN | Inter-alpha-trypsin inhibitor heavy chain H4 | 559.79 | 2 | 460 | 470 | 52 | 3.2.E-02 | GSEMVVAGKLQ |  |
| ITIH4_HUMAN | Inter-alpha-trypsin inhibitor heavy chain H4 | 410.23 | 3 | 606 | 616 | 54 | 1.9.E-02 | IPKPEASFSPR |  |
| ITIH4_HUMAN | Inter-alpha-trypsin inhibitor heavy chain H4 | 463.91 | 3 | 460 | 472 | 53 | 3.0.E-02 | GSEMVVAGKLQDR |  |
| ITIH4_HUMAN | Inter-alpha-trypsin inhibitor heavy chain H4 | 469.24 | 3 | 460 | 472 | 57 | 1.2.E-02 | GSEMVVAGKLQDR | Oxidation (M) |
| K1C10_HUMAN | Keratin, type I cytoskeletal 10 | 595.30 | 2 | 295 | 305 | 66 | 1.3.E-03 | RNVSTGDVNVE |  |
| K1C16_HUMAN | Keratin, type I cytoskeletal 16 | 630.78 | 2 | 442 | 453 | 55 | 1.1.E-02 | EVFTSSSSSSSR |  |
| K1C9_HUMAN | Keratin, type I cytoskeletal 9 | 604.96 | 3 | 12 | 32 | 57 | 1.1.E-02 | SRSGGGGGGGLGSGGSIRSSY |  |
| K2018_HUMAN | Basic helix-loop-helix domain-containing protein KIAA2018 | 573.80 | 2 | 367 | 379 | 56 | 1.2.E-02 | STATVVASSAPGV |  |
| K22E_HUMAN | Keratin, type II cytoskeletal 2 epidermal | 456.23 | 2 | 142 | 151 | 51 | 2.7.E-02 | GPGGYPGGIH |  |
| K22E_HUMAN | Keratin, type II cytoskeletal 2 epidermal | 588.63 | 3 | 44 | 63 | 63 | 2.8.E-03 | SRHGGGGGGFGGGGFGSRSL |  |
| K2C1_HUMAN | Keratin, type II cytoskeletal 1 | 693.97 | 3 | 589 | 616 | 69 | 4.8.E-04 | GGSGGGGGGSSGGRGSGGGSSGGSIGGR |  |
| K2C5_HUMAN | Keratin, type II cytoskeletal 5 | 705.86 | 2 | 16 | 29 | 69 | 6.0.E-04 | SFSTASAITPSVSR |  |
| K2C6A_HUMAN | Keratin, type II cytoskeletal 6A | 506.75 | 2 | 31 | 40 | 70 | 3.7.E-04 | SGFSSVSVSR |  |
| K2C6A_HUMAN | Keratin, type II cytoskeletal 6A | 805.92 | 2 | 534 | 551 | 88 | 9.0.E-06 | AIGGGLSSVGGGSSTIKY |  |
| K2C6A_HUMAN | Keratin, type II cytoskeletal 6A | 806.74 | 3 | 534 | 559 | 69 | 8.5.E-04 | AIGGGLSSVGGGSSTIKYTTTSSSSR |  |
| K2C6C_HUMAN | Keratin, type II cytoskeletal 6C | 513.75 | 2 | 31 | 40 | 63 | 1.8.E-03 | SGFSSISVSR |  |
| KLRAQ_HUMAN | KLRAQ motif-containing protein 1 | 571.35 | 2 | 630 | 640 | 55 | 7.5.E-03 | EATAKAVLEPI |  |
| KNG1_HUMAN | Kininogen-1 | 436.16 | 2 | 462 | 468 | 53 | 3.8.E-03 | DDDLEHQ |  |
| KNG1_HUMAN | Kininogen-1 | 561.72 | 2 | 462 | 471 | 57 | 2.6.E-03 | DDDLEHQGGH |  |
| KNG1_HUMAN | Kininogen-1 | 529.56 | 3 | 462 | 475 | 52 | 1.7.E-02 | DDDLEHQGGHVLDH |  |
| KNG1_HUMAN | Kininogen-1 | 737.02 | 3 | 420 | 438 | 51 | 4.1.E-02 | KHNLGHGHKHERDQGHGHQ |  |
| KV101_HUMAN | Ig kappa chain V-I region AG | 690.80 | 2 | 1 | 13 | 56 | 8.0.E-03 | DIQMTQSPSSLSA | Oxidation (M) |
| KV114_HUMAN | Ig kappa chain V-I region OU | 764.73 | 3 | 1 | 22 | 51 | 5.6.E-02 | DIQMTQSPSSLSASVGNRVTIT |  |
| KV302_HUMAN | Ig kappa chain V-III region SIE | 679.39 | 2 | 1 | 13 | 48 | 5.8.E-02 | EIVLTQSPGTLSL |  |
| KV401_HUMAN | Ig kappa chain V-IV region (Fragment) | 773.08 | 3 | 1 | 22 | 54 | 2.5.E-02 | DIVMTQSPDSLAVSLGERATIN |  |
| KYNU_HUMAN | Kynureninase | 501.75 | 2 | 90 | 97 | 52 | 2.8.E-02 | ELDKWAKI |  |
| LAC_HUMAN | Ig lambda chain C regions | 676.32 | 2 | 93 | 105 | 54 | 1.7.E-02 | STVEKTVAPTECS |  |
| LBN_HUMAN | Limbin | 453.26 | 2 | 306 | 313 | 52 | 2.2.E-02 | FLLSLVLT |  |
| LIRA1_HUMAN | Leukocyte immunoglobulin-like receptor subfamily A member 1 | 571.35 | 2 | 449 | 460 | 47 | 4.7.E-02 | MGIAGLVLVVLG |  |
| MACD2_HUMAN | MACRO domain-containing protein 2 | 479.76 | 2 | 280 | 289 | 49 | 5.8.E-02 | DGVNTVTVPG |  |
| MAGI3_HUMAN | Membrane-associated guanylate kinase, WW and PDZ domain-containing protein 3 | 551.76 | 2 | 247 | 257 | 51 | 3.2.E-02 | AINGSGNAENR |  |
| MOES_HUMAN | Moesin | 548.61 | 3 | 392 | 405 | 58 | 8.6.E-03 | ERQEAEEAKEALLQ |  |
| MYADM_HUMAN | Myeloid-associated differentiation marker | 696.38 | 2 | 2 | 14 | 52 | 2.9.E-02 | PVTVTRTTITTTT |  |
| NACC1_HUMAN | Nucleus accumbens-associated protein 1 | 487.77 | 2 | 61 | 69 | 52 | 2.9.E-02 | NSRSAVVEL |  |
| NEUG_HUMAN | Neurogranin | 626.31 | 2 | 59 | 75 | 79 | 6.2.E-05 | GGPGGAGVARGGAGGGP |  |
| NEUG_HUMAN | Neurogranin | 563.31 | 3 | 54 | 75 | 51 | 4.5.E-02 | KGPGPGGPGGAGVARGGAGGGP |  |
| NPC1_HUMAN | Niemann-Pick C1 protein | 477.18 | 2 | 979 | 986 | 44 | 3.7.E-02 | MFLSDNPN | Oxidation (M) |
| PARK7_HUMAN | Protein DJ-1 | 551.30 | 2 | 93 | 101 | 51 | 3.6.E-02 | KEQENRKGL |  |
| PDLI1_HUMAN | PDZ and LIM domain protein 1 | 625.96 | 3 | 221 | 237 | 55 | 1.3.E-02 | EILESEEKGDPNKPSGF |  |
| PKP4_HUMAN | Plakophilin-4 | 488.24 | 2 | 889 | 898 | 58 | 7.8.E-03 | VSSGATALRN |  |
| PMS1_HUMAN | PMS1 protein homolog 1 | 539.25 | 2 | 100 | 109 | 55 | 1.3.E-02 | ICCIAEVLIT |  |
| PRSS8_HUMAN | Prostasin | 507.29 | 2 | 297 | 306 | 51 | 2.8.E-02 | FLPLGLALGL |  |
| PXDC2_HUMAN | Plexin domain-containing protein 2 | 543.59 | 3 | 46 | 60 | 67 | 8.8.E-04 | DTNRASVGQDSPEPR |  |
| RASF8_HUMAN | Ras association domain-containing protein 8 | 579.29 | 2 | 303 | 312 | 49 | 5.6.E-02 | DIQGQQSLRL |  |
| RHBT2_HUMAN | Rho-related BTB domain-containing protein 2 | 453.23 | 2 | 399 | 406 | 49 | 5.8.E-02 | EMAEDPLT |  |
| RHN2L_HUMAN | Putative rhophilin-2-like protein | 635.38 | 2 | 21 | 32 | 46 | 5.3.E-02 | EAFTVPLIPLGL |  |
| SAA_HUMAN | Serum amyloid A protein | 659.32 | 2 | 69 | 81 | 52 | 3.1.E-02 | FGHGAEDSLADQA |  |
| SAA_HUMAN | Serum amyloid A protein | 937.88 | 2 | 69 | 86 | 88 | 3.2.E-06 | FGHGAEDSLADQAANEWG |  |
| SDPR_HUMAN | Serum deprivation-response protein | 623.77 | 2 | 348 | 359 | 76 | 8.2.E-05 | EGEIAEEAAEKA |  |
| SDPR_HUMAN | Serum deprivation-response protein | 674.29 | 2 | 348 | 360 | 48 | 5.1.E-02 | EGEIAEEAAEKAT |  |
| SDPR_HUMAN | Serum deprivation-response protein | 723.83 | 2 | 347 | 360 | 61 | 3.0.E-03 | VEGEIAEEAAEKAT |  |
| SDPR_HUMAN | Serum deprivation-response protein | 780.37 | 2 | 346 | 360 | 51 | 3.5.E-02 | LVEGEIAEEAAEKAT |  |
| SDPR_HUMAN | Serum deprivation-response protein | 573.28 | 3 | 344 | 360 | 50 | 4.8.E-02 | SALVEGEIAEEAAEKAT |  |
| SDPR_HUMAN | Serum deprivation-response protein | 673.34 | 2 | 347 | 359 | 80 | 5.9.E-05 | VEGEIAEEAAEKA |  |
| SDPR_HUMAN | Serum deprivation-response protein | 767.38 | 2 | 347 | 361 | 51 | 4.0.E-02 | VEGEIAEEAAEKATS |  |
| SDPR_HUMAN | Serum deprivation-response protein | 768.34 | 2 | 309 | 322 | 50 | 3.8.E-02 | AENETKSEDLPSSE |  |
| SDPR_HUMAN | Serum deprivation-response protein | 615.30 | 3 | 178 | 194 | 53 | 3.1.E-02 | GAVEGKEELPDENKSLE |  |
| SRGN_HUMAN | Serglycin | 546.91 | 3 | 110 | 124 | 58 | 5.6.E-03 | DRNLPSDSQDLGQHG |  |
| SRGN_HUMAN | Serglycin | 613.61 | 3 | 108 | 124 | 49 | 4.5.E-02 | SLDRNLPSDSQDLGQHG |  |
| SRGN_HUMAN | Serglycin | 927.38 | 2 | 112 | 128 | 56 | 6.3.E-03 | NLPSDSQDLGQHGLEED |  |
| SRGN_HUMAN | Serglycin | 775.68 | 3 | 108 | 128 | 57 | 8.3.E-03 | SLDRNLPSDSQDLGQHGLEED |  |
| SRGN_HUMAN | Serglycin | 873.71 | 3 | 108 | 130 | 65 | 1.0.E-03 | SLDRNLPSDSQDLGQHGLEEDFM | Oxidation (M) |
| SRGN_HUMAN | Serglycin | 868.40 | 3 | 108 | 130 | 53 | 2.5.E-02 | SLDRNLPSDSQDLGQHGLEEDFM |  |
| STK3_HUMAN | Serine/threonine-protein kinase 3 | 419.17 | 2 | 210 | 217 | 60 | 2.5.E-03 | TSIEMAEG |  |
| TAGL2_HUMAN | Transgelin-2 | 821.91 | 2 | 163 | 177 | 58 | 9.7.E-03 | SDNQLQEGKNVIGLQ |  |
| TBA4A_HUMAN | Tubulin alpha-4A chain | 592.34 | 2 | 361 | 372 | 56 | 9.8.E-03 | TVVPGGDLAKVQ |  |
| TBA4A_HUMAN | Tubulin alpha-4A chain | 650.35 | 2 | 69 | 79 | 66 | 1.0.E-03 | DLEPTVIDEIR |  |
| TBA4A_HUMAN | Tubulin alpha-4A chain | 768.86 | 2 | 245 | 258 | 64 | 2.0.E-03 | DGALNVDLTEFQTN |  |
| TBB1_HUMAN | Tubulin beta-1 chain | 815.37 | 3 | 431 | 451 | 52 | 3.1.E-02 | VLEEDEEVTEEAEMEPEDKGH |  |
| TBB1_HUMAN | Tubulin beta-1 chain | 744.64 | 3 | 433 | 451 | 71 | 1.4.E-04 | EEDEEVTEEAEMEPEDKGH |  |
| TBB4_HUMAN | Tubulin beta-4 chain | 809.11 | 3 | 168 | 190 | 51 | 4.3.E-02 | SVVPSPKVSDTVVEPYNATLSVH |  |
| THRB_HUMAN | Prothrombin | 553.77 | 2 | 322 | 330 | 59 | 6.0.E-03 | SLEDKTERE |  |
| THRB_HUMAN | Prothrombin | 546.94 | 3 | 318 | 330 | 55 | 1.6.E-02 | FEKKSLEDKTERE |  |
| THRB_HUMAN | Prothrombin | 904.37 | 2 | 273 | 289 | 56 | 5.0.E-03 | EETGDGLDEDSDRAIEG |  |
| THRB_HUMAN | Prothrombin | 651.66 | 3 | 324 | 339 | 53 | 3.1.E-02 | EDKTERELLESYIDGR |  |
| THRB_HUMAN | Prothrombin | 718.35 | 3 | 322 | 339 | 50 | 5.4.E-02 | SLEDKTERELLESYIDGR |  |
| THRB_HUMAN | Prothrombin | 781.36 | 2 | 291 | 303 | 76 | 1.1.E-04 | TATSEYQTFFNPR |  |
| THRB_HUMAN | Prothrombin | 608.65 | 3 | 325 | 339 | 52 | 3.3.E-02 | DKTERELLESYIDGR |  |
| THRB_HUMAN | Prothrombin | 638.78 | 2 | 278 | 289 | 50 | 3.7.E-02 | GLDEDSDRAIEG |  |
| TMSL1_HUMAN | Thymosin beta-4-like protein 1 | 705.03 | 3 | 26 | 44 | 61 | 5.1.E-03 | KNPLPSKETIEQEKQAGES |  |
| TMSL1_HUMAN | Thymosin beta-4-like protein 1 | 708.12 | 4 | 20 | 44 | 55 | 2.1.E-02 | KTETQEKNPLPSKETIEQEKQAGES |  |
| TTHY_HUMAN | Transthyretin | 479.76 | 2 | 119 | 127 | 60 | 5.1.E-03 | TAVVTNPKE |  |
| TTHY_HUMAN | Transthyretin | 530.27 | 2 | 118 | 127 | 51 | 4.2.E-02 | TTAVVTNPKE |  |
| TTHY_HUMAN | Transthyretin | 573.79 | 2 | 117 | 127 | 50 | 5.0.E-02 | STTAVVTNPKE |  |
| TTHY_HUMAN | Transthyretin | 655.31 | 2 | 116 | 127 | 75 | 1.3.E-04 | YSTTAVVTNPKE |  |
| TTHY_HUMAN | Transthyretin | 698.82 | 2 | 115 | 127 | 73 | 1.9.E-04 | SYSTTAVVTNPKE |  |
| TTHY_HUMAN | Transthyretin | 780.35 | 2 | 114 | 127 | 61 | 2.6.E-03 | YSYSTTAVVTNPKE |  |
| TTHY_HUMAN | Transthyretin | 543.59 | 3 | 89 | 103 | 67 | 8.6.E-04 | EHAEVVFTANDSGPR |  |
| TTHY_HUMAN | Transthyretin | 872.40 | 2 | 112 | 127 | 86 | 1.2.E-05 | SPYSYSTTAVVTNPKE |  |
| TTHY_HUMAN | Transthyretin | 589.28 | 3 | 88 | 103 | 69 | 5.6.E-04 | HEHAEVVFTANDSGPR |  |
| TTHY_HUMAN | Transthyretin | 657.34 | 3 | 110 | 127 | 55 | 1.8.E-02 | LLSPYSYSTTAVVTNPKE |  |
| TTHY_HUMAN | Transthyretin | 830.43 | 3 | 105 | 127 | 60 | 6.6.E-03 | YTIAALLSPYSYSTTAVVTNPKE |  |
| TTHY_HUMAN | Transthyretin | 882.46 | 3 | 104 | 127 | 57 | 1.3.E-02 | RYTIAALLSPYSYSTTAVVTNPKE |  |
| TTHY_HUMAN | Transthyretin | 790.14 | 4 | 99 | 127 | 54 | 2.8.E-02 | DSGPRRYTIAALLSPYSYSTTAVVTNPKE |  |
| TTHY_HUMAN | Transthyretin | 898.48 | 4 | 95 | 127 | 53 | 4.0.E-02 | FTANDSGPRRYTIAALLSPYSYSTTAVVTNPKE |  |
| TTHY_HUMAN | Transthyretin | 761.42 | 4 | 100 | 127 | 62 | 4.1.E-03 | SGPRRYTIAALLSPYSYSTTAVVTNPKE |  |
| VASP_HUMAN | Vasodilator-stimulated phosphoprotein | 785.04 | 3 | 282 | 302 | 71 | 4.7.E-04 | EKTPKDESANQEEPEARVPAQ |  |
| VTDB_HUMAN | Vitamin D-binding protein | 622.82 | 2 | 448 | 458 | 51 | 4.2.E-02 | SEIDAELKNIL |  |
| VTNC_HUMAN | Vitronectin | 855.41 | 2 | 100 | 116 | 62 | 2.9.E-03 | EEEAPAPEVGASKPEGI |  |
| ZYX_HUMAN | Zyxin | 791.43 | 2 | 348 | 361 | 71 | 3.6.E-04 | PGPLTLKEVEELEQ |  |
