# Supplementary material for: A Comprehensive Peptidome Profiling Technology for the Identification of Early Detection Biomarkers for Lung Adenocarcinoma
Source: PLoS One. 2011 Apr 12;6(4):e18567. doi: 10.1371/journal.pone.0018567 (PMC3075260; doi:10.1371/journal.pone.0018567)
Supplement: Table S3 — (DOC) [file pone.0018567.s006.doc]

**Table S3**

*The MRM channels used in the optimization of transitions.*

| **Clusters** | **Protein ID** | **Q1** | **Q3** | **Dwell** | **CE** | **^a^Rank** | **Sequence** |
| --- | --- | --- | --- | --- | --- | --- | --- |
| **Cluster_00126** | FIBA_7_15 | 432.7 | 235.10 | 20 | 25.0 | 1 | DFLAEGGGV |
|  |  |  | 263.09 | 20 | 25.0 | 2 |  |
|  |  |  | 175.09 | 20 | 25.0 | 3 |  |
|  |  |  | 118.07 | 20 | 25.0 | 4 |  |
| **Cluster_00159** | FIBA_7_16 | 510.74 | 235.10 | 20 | 28.5 | 1 | DFLAEGGGVR |
|  |  |  | 263.09 | 20 | 28.5 | 2 |  |
|  |  |  | 645.32 | 20 | 28.5 | 3 |  |
|  |  |  | 574.28 | 20 | 28.5 | 4 |  |
| **Cluster_00166** | FIBA_5_15 | 525.73 | 175.10 | 20 | 29.1 | 1 | EGDFLAEGGGV |
|  |  |  | 72.08 | 20 | 29.1 | 2 |  |
|  |  |  | 489.21 | 20 | 29.1 | 3 |  |
|  |  |  | 118.08 | 20 | 29.1 | 4 |  |
| **Cluster_00174** | FIBA_6_16 | 539.27 | 645.32 | 20 | 29.7 | 1 | GDFLAEGGGVR |
|  |  |  | 574.28 | 20 | 29.7 | 2 |  |
|  |  |  | 758.39 | 20 | 29.7 | 3 |  |
|  |  |  | 445.23 | 20 | 29.7 | 4 |  |
| **Cluster_00180** | FIBA_4_15 | 554.25 | 489.22 | 20 | 30.4 | 1 | GEGDFLAEGGGV |
|  |  |  | 175.11 | 20 | 30.4 | 2 |  |
|  |  |  | 289.14 | 20 | 30.4 | 3 |  |
|  |  |  | 118.08 | 20 | 30.4 | 4 |  |
| **Cluster_00196** | FIBA_3_15 | 597.76 | 175.10 | 20 | 32.3 | 1 | SGEGDFLAEGGGV |
|  |  |  | 289.14 | 20 | 32.3 | 2 |  |
|  |  |  | 118.08 | 20 | 32.3 | 3 |  |
|  |  |  | 489.22 | 20 | 32.3 | 4 |  |
| **Cluster_00207** | FIBA_4_16 | 632.29 | 645.34 | 20 | 33.8 | 1 | GEGDFLAEGGGVR |
|  |  |  | 445.25 | 20 | 33.8 | 2 |  |
|  |  |  | 758.43 | 20 | 33.8 | 3 |  |
|  |  |  | 574.30 | 20 | 33.8 | 4 |  |
| **Cluster_00221** | FIBA_3_16 | 675.81 | 645.34 | 20 | 35.7 | 1 | SGEGDFLAEGGGVR |
|  |  |  | 758.41 | 20 | 35.7 | 2 |  |
|  |  |  | 445.25 | 20 | 35.7 | 3 |  |
|  |  |  | 574.30 | 20 | 35.7 | 4 |  |
| **Cluster_00240** | FIBA_2_16 | 733.33 | 645.32 | 20 | 38.3 | 1 | DSGEGDFLAEGGGVR |
|  |  |  | 574.28 | 20 | 38.3 | 2 |  |
|  |  |  | 758.39 | 20 | 38.3 | 3 |  |
|  |  |  | 445.23 | 20 | 38.3 | 4 |  |
| **Cluster_00248** | FIBA_1_16 | 768.85 | 645.34 | 20 | 39.8 | 1 | ADSGEGDFLAEGGGVR |
|  |  |  | 445.23 | 20 | 39.8 | 2 |  |
|  |  |  | 574.28 | 20 | 39.8 | 3 |  |
|  |  |  | 758.41 | 20 | 39.8 | 4 |  |
| **Cluster_03342** | FIBA_5_16 | 603.77 | 645.32 | 20 | 32.6 | 1 | EGDFLAEGGGVR |
|  |  |  | 445.26 | 20 | 32.6 | 2 |  |
|  |  |  | 574.28 | 20 | 32.6 | 3 |  |
|  |  |  | 120.07 | 20 | 32.6 | 4 |  |
| **Cluster_02872** | FIBA_6_15 | 461.22 | 320.12 | 20 | 26.3 | 1 | GDFLAEGGGV |
|  |  |  | 289.13 | 20 | 26.3 | 2 |  |
|  |  |  | 175.09 | 20 | 26.3 | 3 |  |
|  |  |  | 120.07 | 20 | 26.3 | 4 |  |
| **Cluster_00135** | LBN_306_313 | 453.23 | 645.32 | 20 | 25.9 | 1 | FLLSLVLT |
|  |  |  | 233.16 | 20 | 25.9 | 2 |  |
|  |  |  | 173.09 | 20 | 25.9 | 3 |  |
|  |  |  | 261.15 | 20 | 25.9 | 4 |  |
| **Cluster_02454** | APOE_194_214 | 756.4 | 173.12 | 20 | 25.8 | 1 | TVGSLAGQPLQERAQAWGERL |
|  |  |  | 1034.01 | 20 | 25.8 | 2 |  |
|  |  |  | 905.43 | 20 | 25.8 | 3 |  |
|  |  |  | 201.12 | 20 | 25.8 | 4 |  |
| **Cluster_03187** | ACCN4_613_624 | 551.76 | 175.09 | 20 | 30.3 | 1 | CPSLGRAEGGGV |
|  |  |  | 72.07 | 20 | 30.3 | 2 |  |
|  |  |  | 118.08 | 20 | 30.3 | 3 |  |
|  |  |  | 129.09 | 20 | 30.3 | 4 |  |
| **Cluster_03444** | APOA4_273_283 | 629.81 | 645.32 | 20 | 33.7 | 1 | GGHLDQQVEEF |
|  |  |  | 445.23 | 20 | 33.7 | 2 |  |
|  |  |  | 758.39 | 20 | 33.7 | 3 |  |
|  |  |  | 574.30 | 20 | 33.7 | 4 |  |
| **Cluster_03498** | APOA4_268_284 | 643.27 | 120.07 | 20 | 34.3 | 1 | SLAELGGHLDQQVEEFR |
|  |  |  | 177.08 | 20 | 34.3 | 2 |  |
|  |  |  | 120.08 | 20 | 34.3 | 3 |  |
|  |  |  | 645.35 | 20 | 34.3 | 4 |  |
| **Cluster_03661** | APOA4_260_284 | 689.78 | 120.07 | 20 | 36.4 | 1 | GNTEGLQKSLAELGGHLDQQVEEFR |
|  |  |  | 177.07 | 20 | 36.4 | 2 |  |
|  |  |  | 593.21 | 20 | 36.4 | 3 |  |
|  |  |  | 722.25 | 20 | 36.4 | 4 |  |
| **Cluster_03858** | APOA4_271_283 | 750.85 | 277.11 | 20 | 39.0 | 1 | ELGGHLDQQVEEF |
|  |  |  | 166.08 | 20 | 39.0 | 2 |  |
|  |  |  | 231.10 | 20 | 39.0 | 3 |  |
|  |  |  | 259.08 | 20 | 39.0 | 4 |  |
| **BSA** | BSA_461.8 | 461.8 | 722.4 | 20 | 29.1 |  |  |
|  | BSA_464.3 | 464.3 | 651.4 | 20 | 29.3 |  |  |
|  | BSA_547.3 | 547.3 | 589.3 | 20 | 32.9 |  |  |
|  | BSA_582.3 | 582.3 | 951.5 | 20 | 34.4 |  |  |
|  | BSA_653.4 | 653.4 | 1055.6 | 20 | 38 |  |  |

*^a^* The numbers indicate the 1^st^, 2^nd^, 3^rd^, or 4^th^ most intense fragment ion in QSTAR-Elite MS/MS spectra.
