# Supplementary material for: A Comprehensive Peptidome Profiling Technology for the Identification of Early Detection Biomarkers for Lung Adenocarcinoma
Source: PLoS One. 2011 Apr 12;6(4):e18567. doi: 10.1371/journal.pone.0018567 (PMC3075260; doi:10.1371/journal.pone.0018567)
Supplement: Table S4 — (DOC) [file pone.0018567.s007.doc]

**Table S4**

*The MRM channel list used in validation experiments.*

| **Peptide ID** | **m/z precursor**  **Q1** | **m/z fragment**  **(Q3)** | **Dwell**  **time (msec)** | **Collision**  **energy** |
| --- | --- | --- | --- | --- |
| ACCN4_613_624 | 551.8 | 175.1 | 25 | 30.3 |
| APOA4_260_284 | 689.8 | 120.1 | 25 | 36.4 |
| APOA4_268_284 | 643.3 | 120.1 | 25 | 34.3 |
| APOA4_271_283 | 750.9 | 259.1 | 25 | 39.0 |
| APOA4_273_283 | 629.8 | 645.3 | 25 | 33.7 |
| APOE_194_214 | 756.4 | 1034.0 | 25 | 25.8 |
| FIBA_1_16 | 768.9 | 645.3 | 25 | 39.8 |
| FIBA_2_16 | 733.3 | 645.3 | 25 | 38.3 |
| FIBA_3_15 | 597.8 | 175.1 | 25 | 32.3 |
| FIBA_3_16 | 675.8 | 645.3 | 25 | 35.7 |
| FIBA_4_15 | 554.3 | 175.1 | 25 | 30.4 |
| FIBA_4_16 | 632.3 | 645.3 | 25 | 33.8 |
| FIBA_5_15 | 525.7 | 175.1 | 25 | 29.1 |
| FIBA_5_16 | 603.8 | 645.3 | 25 | 32.6 |
| FIBA_6_15 | 461.2 | 120.1 | 25 | 26.3 |
| FIBA_6_16 | 539.3 | 645.3 | 25 | 29.7 |
| FIBA_7_15 | 432.7 | 235.1 | 25 | 25.0 |
| FIBA_7_16 | 510.7 | 645.3 | 25 | 28.5 |
| LBN_306_313 | 453.2 | 645.3 | 25 | 25.9 |
| *^a^* BSA | 461.8 | 722.4 | 25 | 29.1 |
| *^a^* BSA | 464.3 | 651.4 | 25 | 29.3 |
| *^a^* BSA | 547.3 | 589.3 | 25 | 32.9 |
| *^a^* BSA | 582.3 | 951.5 | 25 | 34.4 |
| *^a^* BSA | 653.4 | 1055.6 | 25 | 38 |

*^a^* 5 fragments of digested BSA were used for data normalization.
